# Identification and Analysis of Zinc Efficiency-Associated Loci in Maize

**DOI:** 10.3389/fpls.2021.739282

**Published:** 2021-11-15

**Authors:** Jianqin Xu, Xuejie Wang, Huaqing Zhu, Futong Yu

**Affiliations:** Key Laboratory of Plant-Soil Interaction (MOE), Centre for Resources, Environment and Food Security, College of Resources and Environmental Sciences, China Agricultural University, Beijing, China

**Keywords:** maize (*Zea mays* L.), quantitative trait locus (QTL), zinc (Zn) deficiency tolerance, ZRT/IRT-like protein (ZIP), candidate genes

## Abstract

Zinc (Zn) deficiency, a globally predominant micronutrient disorder in crops and humans, reduces crop yields and adversely impacts human health. Despite numerous studies on the physiological mechanisms underlying Zn deficiency tolerance, its genetic basis of molecular mechanism is still poorly understood. Thus, the Zn efficiency of 20 maize inbred lines was evaluated, and a quantitative trait locus (QTL) analysis was performed in the recombination inbred line population derived from the most Zn-efficient (Ye478) and Zn-inefficient inbred line (Wu312) to identify the candidate genes associated with Zn deficiency tolerance. On this basis, we analyzed the expression of *ZmZIP1*-*ZmZIP8*. Thirteen QTLs for the traits associated with Zn deficiency tolerance were detected, explaining 7.6–63.5% of the phenotypic variation. The genes responsible for Zn uptake and transport across membranes (*ZmZIP3*, *ZmHMA3*, *ZmHMA4*) were identified, which probably form a sophisticated network to regulate the uptake, translocation, and redistribution of Zn. Additionally, we identified the genes involved in the indole-3-acetic acid (IAA) biosynthesis (*ZmIGPS*) and auxin-dependent gene regulation (*ZmIAA*). Notably, a high upregulation of *ZmZIP3* was found in the Zn-deficient root of Ye478, but not in that of Wu312. Additionally, *ZmZIP4*, *ZmZIP5*, and *ZmZIP7* were up-regulated in the Zn-deficient roots of Ye478 and Wu312. Our findings provide a new insight into the genetic basis of Zn deficiency tolerance.

## Introduction

Maize (*Zea mays* L.) is the third most important cereal crop globally, after wheat and rice, and is a foundational model for genetics and genomics (Jiao et al., 2017). It accounts for about 64% of coarse grain and 27.1% of the total cereal production, providing at least 30% of food calories to more than 4.5 billion people in 94 developing countries (Shiferaw et al., 2011; Wu and Guclu, 2013). At present, China and other rapidly developing economies face the challenge of substantially increasing the yield of cereal grains (Chen et al., 2011). China must increase its agricultural output by 50% to meet its growing food demand in the future (Cui et al., 2014). China is the second-largest producer of maize after the United States (Xomphoutheb et al., 2020). Maize is an important crop to solve the cereal demand in China.

Zinc is an essential element in the range of physiological and biochemical mechanisms of plants and is needed for more than 300 plant enzymes, and functions as co-factors in the processes of photosynthesis, respiration, and other metabolic reactions. Within the broad category of mineral-related abiotic stresses, Zn deficiency is one of the most widespread limiting factors to crop production, affecting 50% of cereal crops soil (Nielsen, 2012). Zn deficiency in plants leads to leaf bronzing, stunted growth, shortened internodes and petioles, malformed leaves, delayed flowering and fruit maturity, spikelet sterility, and seedling mortality (Widodo et al., 2010; Gainza-Cortés et al., 2012; Mattiello et al., 2015), thus reducing the yield and quality of crops (Broadley et al., 2007; Graham et al., 2012). Zn is not only an important trace element for plants but also an indispensable nutrient for humans. As the second most abundant micronutrient, Zn serves as a structural component of at least 3,000 proteins in the body (Sasaki et al., 2018). Zn deficiency causes a variety of diseases (Kambe et al., 2015; King et al., 2015), which in the worst case may cause human death (Wessells and Brown, 2012). The WHO estimates that one-third of the world population suffers from Zn shortage, especially in developing countries (Swain et al., 2016). Therefore, understanding the mechanisms of Zn deficiency tolerance would be helpful to improve the tolerance of maize to Zn deficiency via conventional breeding using marker assistant selection or through transgenic technology.

The ability to tolerate Zn deficiency can also be termed Zn efficiency (ZE), which is evaluated by the ability of a plant to grow and yield well under Zn-deficient conditions (Impa et al., 2013a). Studies on the physiological mechanisms underlying ZE have been reported in several other crop species, including beans (Hacisalihoglu et al., 2001), barley (Genc et al., 2007), wheat (Rengel and Graham, 1996), and rice (Wissuwa et al., 2006). However, the mechanisms of ZE in maize are still unclear. Several studies implicated that some root processes could increase the bioavailability of soil Zn for root uptake to enhance ZE, mainly including the release of root exudates (Khoshgoftarmanesh et al., 2018) and morphological changes in roots (Mori et al., 2016). In addition to Zn uptake, ZE is probably related to root-to-shoot transport and the remobilization of Zn from old to young leaves (Impa et al., 2013b). Besides, the shoot-localized mechanism in ZE illustrated by Hacisalihoglu et al. (2003), contains the subcellular compartmentation of Zn in shoot cells and the biochemical utilization of Zn in the cells of shoots (Hacisalihoglu and Kochian, 2003; White and Broadley, 2011).

Among these physiological processes, increased Zn uptake from the soil, as a major physiological mechanism, requires Zn transporters which can transport Zn^2+^ across membranes (Kambe et al., 2004). These transporters get involved in the uptake of Zn from the soil, translocation inside plants, loading and unloading of xylem, sequestration in the vacuolar, and remobilization from the vacuole. To date, several metal transporters have been identified in plants, including zinc-regulated transporters, iron-regulated transporters-like protein (ZIP) family, heavy metal ATPases (HMA) family, natural resistance-associated macrophage protein (NRAMP) family, and cation diffusion facilitator (CDF) family. The regulation of the *ZIP* gene family is considered to be a major mechanism in tolerating Zn deficiency stress. Expression analysis showed that the transcripts of *AtZIP1*–*AtZIP5*, *AtZIP9*–*AtZIP12* increased under Zn deficiency, suggesting that these genes may enhance Zn acquisition under Zn-deficient conditions in *Arabidopsis* (Krämer et al., 2007). AtIRT3 reverses growth defects in the Zn- and Fe-uptake-deficient yeast mutants, and the overexpression of *AtIRT3* enhances the Zn accumulation in shoots (Lin et al., 2009). *OsZIP1*, *OsZIP2*, *OsZIP4*, *OsZIP5*, *OsZIP6*, *OsZIP7*, and *OsZIP8* are induced in Zn-deficient roots, and function in the uptake, transport, or allocation of Zn in rice under Zn-deficient conditions (Chen et al., 2008; Lee and An, 2009; Kavitha et al., 2015; Sasaki et al., 2015). Six *HvZIP* genes (*HvZIP3*, *HvZIP5*, *HvZIP7*, *HvZIP8*, *HvZIP10*, and *HvZIP13*) in barley are highly induced in Zn-deficient roots, and their increased expression enhances the uptake and root-to-shoot translocation of Zn in response to Zn deficiency stress (Tiong et al., 2015).

Quantitative trait locus (QTL) analysis is a powerful method to identify the chromosomal regions encompassing genes controlling complex quantitative traits (Alonso-Blanco et al., 2009; Ueno et al., 2009). However, the genetic basis of ZE remains largely unknown, since most studies on QTL identification mainly focus on seed Zn concentration and content (Gu et al., 2015; Jin et al., 2015; Hindu et al., 2018) and only a few researchers have reported QTLs associated with performance under Zn deficiency. Wissuwa et al. (2006) reported the QTLs associated with the most severe and susceptible Zn deficiency symptoms of rice in the field, including leaf bronzing, plant mortality, and biomass reduction in Zn-deficient conditions. Similarly, Genc et al. (2009) have assessed the severity of Zn deficiency symptoms on a scale from 1 to 9 in wheat. The QTLs controlling the Zn score, shoot dry weight, and concentrations of Zn and Fe in shoots and grains were detected at two levels of Zn. By combining bi-parental QTL mapping and genome-wide association analysis, Lee et al. (2017) identified one putative candidate gene which was associated with grain yield and component traits, and confirm that the candidate gene *Os06g44220* is strongly up-regulated in both Zn-deficient root and shoot.

Therefore, the aims of this study are to (1) select the most and least Zn-efficient maize inbred lines for further studies in the linkage analysis and candidate gene expressions; (2) reveal possible physiological mechanisms on ZE; (3) detect the QTLs for ZE in the recombinant inbred line (RIL) population derived from the most and least Zn-efficient maize inbred lines and identify the candidate genes in the QTL co-localization; (4) to analyze the expression patterns of the eight *ZmZIP* genes (*ZmZIP1*-*ZmZIP8*) in response to Zn deficiency.

## Materials and Methods

### Plant Material and Experiment Design

#### Experiment 1: Variations in the Zn Efficiency of Twenty Maize Inbred Lines

Twenty maize inbred lines (Ye478, CI7, Yu87-1, DE3, By815, Zheng58, KUI3, B77, SC55, SK, By804, Dan340, Chang7-2, X178, Mo17, Zong3, B73, HuangC, K22, and Wu312) which have generated linkage populations among them, were grown hydroponically in a mixed-crop system under Zn-deficient (-Zn) (0.3 μmol L^–1^ Zn-EDTA) and Zn-sufficient (CK) (10 μmol L^–1^ Zn-EDTA) conditions. Each treatment contained three replications. For each treatment, three replications for the twenty inbred lines were grown hydroponically in a 40 L tank (665 × 410 × 160 mm^3^, length × width × height).

#### Experiment 2: Quantitative Trait Locus Analysis of Zn Efficiency in Maize

The Zn-efficient (Ye478) and -inefficient inbred lines (Wu312) were selected from Experiment 1. A RIL population consisting of 218 lines were derived from Ye478 (female parent) and Wu312 (male parent), as described by Liu J. et al. (2011). The RIL population and their parents were hydroponically grown under -Zn (0.3 μmol L^–1^) and CK (10 μmol L^–1^) conditions. For each treatment, two independent experiments were conducted in randomized complete blocks with three replications each. Fifty-five RILs and two plants for each parent were grown in a 40 L tank. In total, 12 tanks were used for each experiment.

#### Experiment 3: Expression of *ZIP* Genes in Maize

The expression of eight *ZmZIP* genes (*ZmZIP1*–*ZmZIP8*) in the roots of the Zn-efficient (Ye478) and -inefficient inbred lines (Wu312) were analyzed in two treatments, Experiment 1 and 2. Ye478 and Wu312 were grown hydroponically in a mixed-crop system under -Zn (0.3 μmol L^–1^) and CK (10 μmol L^–1^) conditions. Each treatment contained three biological replications. Each biological replication contained two plants for each inbred line. Mixed cropping was performed on four plants in a 3.3-L tank. Three technical replications were performed for each biological replication.

### Plant Culture in Hydroponics

The maize seeds were sterilized for 30 min in a 10% solution of hydrogen peroxide (H_2_O_2_), washed with distilled water, and soaked in saturated calcium sulfate (CaSO_4_) for 10 h, and then germinated on a moist filter paper in the dark at room temperature. Two days later, the germinated seeds were wrapped in a moist filter paper roll and grown. At the stage of two visible leaves, the seedlings were selected and transferred into a 40 L black tank (Experiment 1 and 2) or a 3.3 L tank (Experiment 3). The solution pH was set at 5.5–6. The adjusted Hoagland nutrient solution contained (mmol L^–1^): 0.5 NH_4_NO_3_, 0.5 CaCl_2_, 1.5 Ca(NO_3_)_2_, 0.75 K_2_SO_4_, 0.65 MgSO_4_, 0.1 KCl, 0.25 KH_2_PO_4_, 1.0 × 10^–3^ H_3_BO_3_, 0.35 Fe(II)-EDTA, 8.0 × 10^–3^ CuSO_4_, 1.2 × 10^–2^ MnSO_4_, 4.0 × 10^–5^ (NH_4_)Mo_7_O_24_, and 4.0 × 10^–3^ NiCl. The nutrient solution was renewed every 3 days and aerated by a pump. The maize seedlings were cultured with hydroponics in a growth chamber condition with controlled conditions, namely, 28°C during the 14 h light period from 8:00 to 22:00 and 22°C during the 10 h dark period, the average light intensity was 350 μmol m^–2^ s^–1^ that was measured at the canopy.

### Data Collection

The hydroponic culture experiments were carried out to collect data in Experiments 1–3 at the seedling stage. Experiment 1 was terminated 21 days after transplanting, and all the samples were dried at 75°C till constant weight. The plant height and shoot and root dry weights were measured and the root to shoot (R/S) ratios and ZE were calculated. The concentrations of Zn, iron (Fe), manganese (Mn), and copper (Cu) in the shoots and roots were analyzed using an inductively coupled plasma-atomic emission spectroscopy (ICP-AES). The ZE, relative ratios of R/S efficiency, nutrient contents, uptake efficiency, and relative transport were estimated using the following equations from (1) to (5), respectively.


(1) $$\mathrm{Zn}\mathrm{efficiency}\left(\%\right)=\frac{Dryweight\left(-Zn\right)}{Dryweight\left(CK\right)}$$

(2) $$\mathrm{Relative}\mathrm{ratios}\mathrm{of}\mathrm{root}\text{to}\mathrm{shoot}\mathrm{efficiency}=\frac{\frac{R}{S}ratio\left(-Zn\right)}{\frac{R}{S}ratio\left(CK\right)}$$

(3) Nutrientcontent=nutrientconcentration×dryweight

(4) $$\mathrm{Uptake}\mathrm{efficiency}=\frac{\text{to}\mathrm{tal}\mathrm{nutrient}\mathrm{content}}{\mathrm{root}\mathrm{dry}\mathrm{weight}}$$

(5) $$\mathrm{Relative}\mathrm{transport}=\frac{\mathrm{shoot}\mathrm{nutrient}\mathrm{content}}{\text{to}\mathrm{tal}\mathrm{nutrient}\mathrm{content}}\times 100\%$$

**FIGURE 1:**
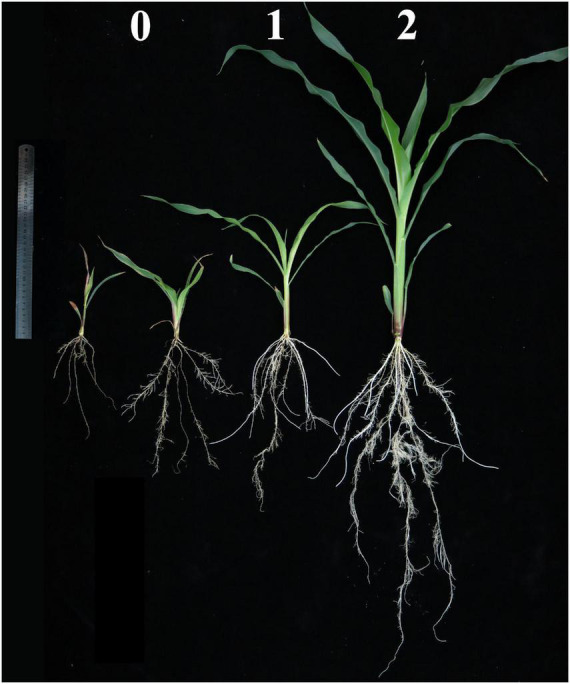
Three scales for Zn score of maize in the RIL population under Zn deficiency. Based on the typical Zn-deficient symptoms in plants on the 15–21th day after transplanting, the Zn score for each plant has been visually recorded three times since the 15th day after transplanting. Three scales (0, 1, 2) are designed to assess the ability of RILs in the Ye478 × Wu312 population to tolerate Zn deficiency under Zn-deficient conditions (0.3 μmol L^−1^ Zn-EDTA). Score-0 plants (two plants on the left) only developed four leaves and showed the severest Zn deficiency symptoms in the shoots, including stunted growth, shortened internodes, small malformed leaves, wrinkled leaf margins and chlorosis and necrotic patches distributed on more than 50% of the areas on all leaves. The root growth of score-0 plants was strongly suppressed, manifested in the great reductions in the root length and number of lateral roots, large-area brown lesions on roots. Compared with the score-0 plants, the score-1 plants showed better growth without brown lesions on roots, but still appeared to have shortened internodes, small malformed leaves and chlorosis distributed on 10–30% of areas on the middle and young leaves. Score 2 represents green healthy plants under Zn-deficient conditions, showing little Zn-deficient symptoms in both shoots and roots when compared with the plants grown in the Zn-sufficient (CK) treatment. The representative shoots and roots are displayed. A ruler of 30 cm length is shown.

Experiment 2 was terminated 21 days after transplanting. All the samples were dried at 75°C over 72 h. The shoot and root dry weights were measured, and the R/S ratio and the ratio of -Zn/CK for each trait were also calculated. Based on the typical Zn deficiency symptoms in plants on the 15–21th day after transplanting, the Zn score for each plant has been visually recorded three times since the 15th day after transplanting. Three scales (0, 1, 2) were designed to assess the tolerance of the RILs in the Ye478 × Wu312 population to Zn deficiency under -Zn conditions (0.3 μmol L^–1^ Zn-EDTA) (Figure 1). The score-0 plants (two plants in the left in Figure 1) only developed four leaves and showed the severest Zn deficiency symptoms in the shoots, including stunted growth, shortened internodes, small malformed leaves, wrinkled leaf margins, and chlorosis and necrotic patches distributed on more than 50% of the areas on all leaves. The root growth of the score-0 plants was strongly suppressed, manifested in the great reductions in the root length and number of lateral roots and the large-area brown lesions on the roots. Compared with the score-0 plants, the score-1 plants showed a better growth without brown lesions on the roots, but still appeared to have shortened internodes, small malformed leaves, and chlorosis distributed on 10–30% of the areas on the middle and young leaves. Score 2 represented the green healthy plants under the Zn-deficient conditions, showing little Zn deficiency symptoms in both the shoots and roots when compared with the plants grown in the CK treatment.

Experiment 3 was terminated 21 days after transplanting. The total RNA was isolated from the roots of Ye478 and Wu312 using TRIzol (Takara, Kusatsu, Shiga, Japan). We used 1.5 μg of the total RNA to synthesize the complementary DNA (cDNA). A quantitative real-time PCR (qRT-PCR) was performed using an SYBR Green Real-time RT-PCR (Applied Biosystems, Waltham, Massachusetts, United States) and an ABI7500 Fast Real-Time PCR System (Applied Biosystems). The primers used for the RT-PCR are shown in Supplementary Table 11.

### Data Analysis

#### Statistical Analysis

In Experiments 1, 2, and 3, the means for each trait were compared using one-way ANOVA at a 0.05 level of probability followed by Turkey’s test using SPSS 20.0 (IBM, Armonk, New York, United States). The linear mixed effect function Imer from the lme4 package in R was fitted to each RIL to obtain the best linear unbiased prediction (BLUP) value for each trait: *yi* = *μ* + *fi* + *ei* + *εi*, where *yi* is the phenotypic value of individual *i*, *μ* is the grand mean for all environments, *fi* is the genetic effect, *ei* is the effect of different environments, and *εi* is the random error. The broad-sense heritability for each trait was calculated by *H*^2^ = *σ_*g*_*^2^/(*σ_*g*_*^2^ + *σ_*ge*_*^2^/*e* + *σ_ε_*^2^/*re*), where *σ_*g*_*^2^ is the genetic variance, *σ_*ge*_*^2^ is the interaction of the genotype and treatment, *σ_ε_*^2^ is the residual error, while *e* and *r* are the number of replications and treatments, respectively. A principal component analysis (PCA) was conducted by CANOCO 4.5. In Experiment 3, the changes in expression were calculated via the ΔΔCt method.

#### Quantitative Trait Locus Mapping

In Experiment 2, a genetic linkage map with a total length of 2,084 cm consisting of 184 polymorphic markers, was constructed by Liu J. et al. (2011). The QTL identification was performed using composite interval mapping (CIM) in the Windows QTL Cartographer version 2.5. Model 6 was selected for detecting the QTLs and estimating their effects. The threshold logarithm of odds (LOD) values needed to declare the putative QTLs were estimated by permutation tests with a minimum of 1,000 replicates at a significant level of *P* < 0.05 (LOD = 3). The confidence interval for each QTL was determined using the 1-LOD interval method.

#### Annotation of Candidate Genes

According to the physical distance of the peak bins, the genes within the refined interval and their functional descriptions were identified using the maizeB73 reference genome assembly version 2 available on the MaizeGDB Genome database^1^.

## Results

### Variations in Zn Efficiency Among Twenty Maize Inbred Lines

#### Effects of Zinc Deficiency on Maize Inbred Lines

**FIGURE 2:**
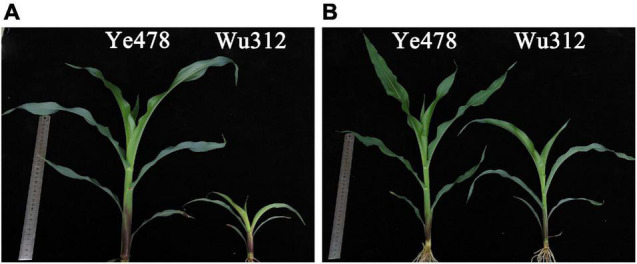
The phenotypes of Ye478 and Wu312 under -Zn and CK conditions. In the -Zn (0.3 μmol L^–1^ Zn-EDTA) **(A)** and CK (10 μmol L^–1^ Zn-EDTA) **(B)** treatments, the maize inbred lines Ye478 (left) and Wu312 (right) were grown hydroponically for 21 days after transplanting. The representative shoots are displayed. Rulers of 30 cm length are shown in both **(A,B)**.

Under Zn-deficient conditions, most maize inbred lines showed chlorosis on young leaves and reduced leaf size. As shown in Figure 2, Wu312 appeared to have the severest symptoms of Zn deficiency, including stunted growth, shortened internodes and petioles, wrinkled leaf margins, and small malformed leaves. However, little difference was observed in Ye478 between the -Zn and CK treatments. Substantial variations in the ZE based on the shoot (Supplementary Figure 1A) and root (Supplementary Figure 1B) dry weights were observed within 20 inbred lines, ranging from 38.5 to 128% and 39.4–146%, respectively. The ZEs based on the shoot and root dry weights of Ye478 and CI7 were the highest, which were about three times as high as those of HuangC, K22, and Wu312.

In general, Zn-efficient genotypes are not only able to accumulate more Zn, but also produce more dry matter weights and grain yields. Our results indicated that Ye478 and CI7 not only showed little Zn deficiency symptoms but also produced higher plant heights (Supplementary Figure 2) and accumulated more shoot and root dry weights in comparison with K22 and Wu312 (Supplementary Table 1). The shoot dry weights of Ye478 and CI7 were more than five times higher than those of K22 and Wu312 under Zn deficiency. The root dry weights of Ye478 and CI7 were more than three times as high as those of K22 and Wu312 under -Zn conditions. No significant differences in the shoot dry weights of Ye478 and CI7 were observed between the -Zn and CK treatments. Zn deficiency markedly enhanced the root dry weights of Ye478 and CI7 by 46 and 32.8%, respectively. By contrast, Zn deficiency significantly decreased the shoot and root dry weights of K22 by 59.9 and 43.7%, and Wu312 by 61.5 and 48.6%, respectively. Additionally, Ye478 and CI7 maintained stable R/S ratios in response to Zn deficiency stress. Low Zn stress significantly increased the R/S ratio of K22 by 33.5%.

**FIGURE 3:**
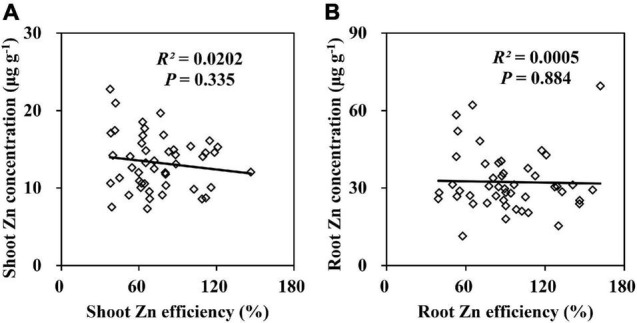
Relationships between Zn efficiencies (ZEs) based on the shoot **(A)** and root **(B)** dry weights and Zn concentrations in the shoot **(A)** and root **(B)** under Zn deficiency. *P* < 0.05 indicates significant differences.

**FIGURE 4:**
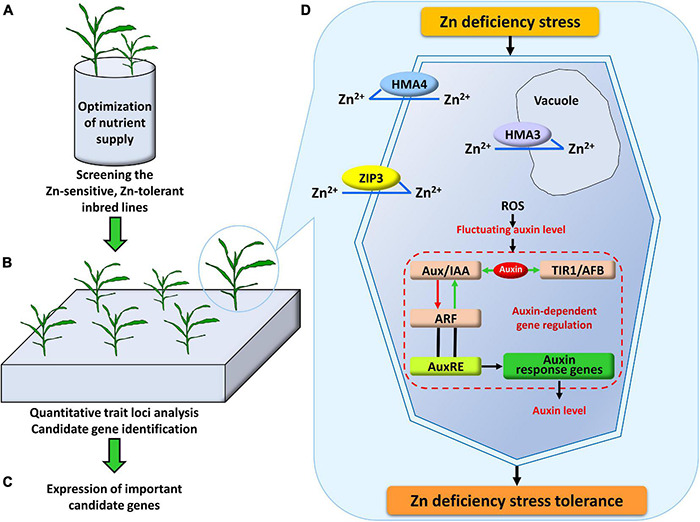
Systematic research design and schematic model for the mechanism of Zn deficiency tolerance in plants. **(A)** The nutrient solution supply was optimized by adjusting the NH_4_^+^/NO_3_^–^ ratio and the supply level of other metal ions. On this basis, the most tolerant and the most sensitive parents under Zn deficiency stress were screened out. **(B)** Quantitative trait loci associated with Zn deficiency tolerance were detected in the linkage population derived from the parents screened out from **(A)**, and candidate genes were identified within these loci. **(C)** Based on the results of physiological experiments and the candidate genes identified by the QTL analysis, the expression of eight ZIP genes (*ZmZIP1*-*8*) was analyzed under -Zn and CK conditions. **(D)** In the mechanisms of Zn deficiency stress tolerance, the important genes which may be involved in the uptake of Zn^2+^ from the soil to roots and preferential distribution to shoot (*ZmZIP3*), sequestering Zn^2+^ into vacuole (*ZmHMA3*), translocating Zn^2+^ from root to shoot (*ZmHMA4*), probably participate in the uptake, transport and redistribution of Zn. In addition, the gene that may repress transcription of ARF plays an essential role in the auxin-dependent gene regulation (*ZmIAA17*).

Zn deficiency decreased the Zn concentrations in the shoots and roots of 20 inbred lines by more than 50% (Supplementary Table 2). Most lines were Zn-deficient plants diagnosed by the shoot Zn concentration which ranged from 7.9 to 20.4 μg g^–1^. A Pearson correlation analysis indicated that the ZE based on the shoot and root dry weights of the 20 inbred lines were not correlated with the Zn concentrations in shoots (Figure 3A) and roots (Figure 3B). Therefore, the Zn concentrations in the shoots and roots were not used in Experiment 2. The Zn accumulation in the shoots and roots was markedly decreased by Zn deficiency. Nevertheless, regardless of the Zn supply, the shoot and root Zn contents of Ye478 and CI7 were remarkably higher than those of K22 and Wu312 (Supplementary Table 3). A greater than eightfold difference in the shoot Zn content occurred between Ye478 (49.9 μg plant^–1^) and Wu312 (6.2 μg plant^–1^). In addition, Zn deficiency resulted in a decrease in the Zn uptake efficiency and an increase in the relative Zn transport of maize inbred lines (Supplementary Table 4). Compared with Wu312, Ye478, and CI7 maintained higher levels of Zn uptake and transport under different conditions. In conclusion, Ye478 and Wu312 were screened out to be the most and least tolerant inbred lines to Zn deficiency, respectively, allowing for further molecular genetic studies in Experiments 2 and 3 (Figure 4A).

In terms of the effects on other mineral elements, Zn deficiency resulted in a 20.1–119.3% increase of Fe concentrations in the shoots (Supplementary Table 5) and a 7.4–60.5% increase of Mn concentrations in the roots (Supplementary Table 6) for most inbred lines. Additionally, much more Mn is retained in the shoots than in roots under Zn-deficient conditions, resulting in the enhancement of the shoot-to-root transport of Mn (Supplementary Table 8). Compared with the CK treatment, higher Cu concentrations were detected in both the shoots and roots in the -Zn treatment (Supplementary Tables 5, 6), which led to the enhancement of Cu uptake and transport (Supplementary Tables 7, 8).

#### Principal Components Analysis of Zn Efficiency

**FIGURE 5:**
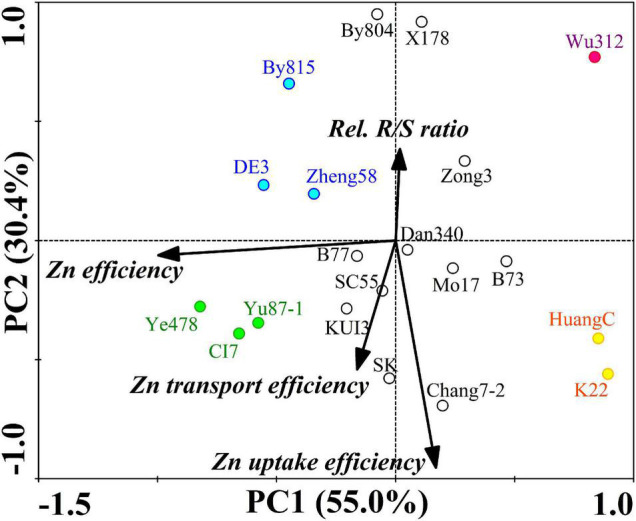
Principal component analysis of ZE based on the shoot dry weight, relative R/S ratio efficiency, Zn uptake efficiency, and Zn transport efficiency (relative Zn transport) under the Zn deficiency of 20 maize inbred lines. Two principal components (PC1 and PC2) were extracted and accounted collectively for 85.4% of the variance. Dots represent the position of the 20 inbred lines determined by their values on the given principal component. The dots in green, blue, orange, and red represent four typical types of maize inbred lines differing in Zn deficiency tolerance. Black lines indicate vectors quantifying the magnitude and direction of a trait’s contribution to the principal component.

The PCA extracted two principal components that accounted collectively for 85.4% of the variance in ZE, relative R/S ratio efficiency, Zn uptake efficiency, and Zn transport efficiency under the Zn deficiency of 20 inbred lines (Figure 5). PC1 was negatively loaded with ZE while PC2 was positively loaded with relative R/S ratio efficiency and negatively loaded with Zn uptake and transport efficiency.

Four typical types of inbred lines differing in ZE are shown in Figure 5. The most Zn-efficient inbred lines with the highest ZEs (109.3–128%), containing Ye478, CI7, and Yu87-1, were efficient in Zn uptake and transport, maintaining stable R/S ratios. Compared with Ye478, CI7, and Yu87-1, the CK inbred lines with lower ZEs (91.6–102.1%), including DE3, By815, and Zheng58, kept lower Zn uptake and transport efficiency as well as higher R/S ratio efficiency. The least Zn-efficient inbred line was Wu312 with the lowest ZE (38.5%) and the lowest Zn uptake and transport. Compared with Wu312, the -Zn inbred lines HuangC and K22, with higher ZEs (39.0–40.3%), had higher efficiency in Zn uptake and transport.

### Quantitative Trait Locus Analysis of Zn Efficiency in Maize

#### Phenotypic Data Analysis

**FIGURE 6:**
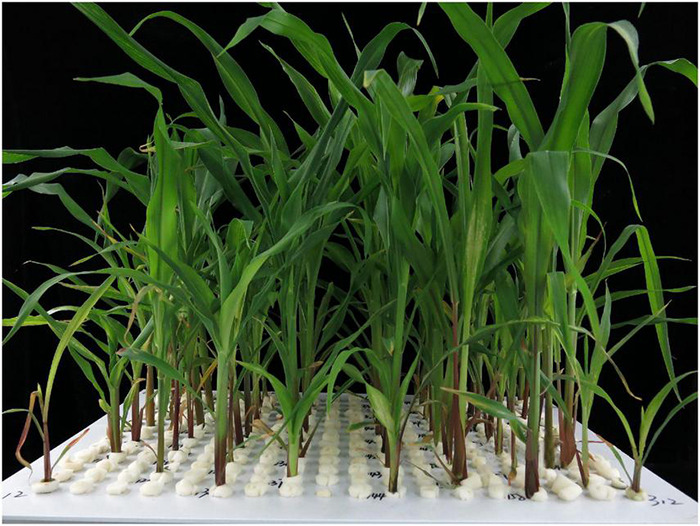
Phenotypic variation in the recombinant inbred line (RIL) population. In the -Zn treatment (0.3 μmol L^–1^ Zn-EDTA), two plants for each parent and 55 RILs were grown in hydroponics for 21 days after transplanting in each tank. Large phenotypic variations of Zn-deficient symptoms were observed among RILs under Zn deficiency.

A RIL population consisting of 218 lines derived from Wu312 and Ye478 was used to identify the Zn efficiency-associated loci (*ZEAL*s) in maize. Consistent with the results of Experiment 1, Wu312 showed the severest Zn-deficient symptoms like the score-0 plants shown in Figure 1, and Ye478 showed green healthy plants without symptoms of Zn deficiency similar with the score-2 plant. Large phenotypic variations were observed in Zn-deficient symptoms among different RILs (Figure 6).

Phenotypic data were collected for each trait from the RILs in two experiments under different conditions (-Zn, CK, -Zn/CK). The phenotypic means and range of each trait for the parents and their RIL population are in Table 1. Our results indicated that Zn deficiency resulted in marked increases in the shoot and root dry weights of Ye478. On the contrary, Zn deficiency significantly decreased the shoot and root dry weights for Wu312 by 60.3 and 50%, respectively. Zn deficiency increased the R/S ratios for Ye478 and Wu312 by 18.2 and 25.0%, respectively. Each physiological trait displayed normal distribution based on the physiological traits among the RILs, and the coefficients of variation (CV) for each trait ranged from 17.4 to 60.8% (Table 1). In the -Zn and -Zn/CK treatments, the means for each trait of the population were between two parents. The broad-sense heritabilities (*H*^2^) of traits were higher than 75%, indicating that traits associated with Zn deficiency tolerance were highly controlled by heredity.

**TABLE 1:** Statistical analysis of phenotypic variation of parents and their RIL population.

**Trait**	**Treatment**	**Parents**		***a***	**RIL population**			
		**Ye478**	**Wu312**		**Mean**	**Range**	**CV (%)**	***H*^2^ (%)**
ZnSc	-Zn	2	0	**	1.5	0–2	52.5	99.6
SDW	-Zn	2.38	0.48	**	0.91	0.16–2.7	60.8	94.1
	CK	2.23	1.21	**	1.59	0.47–3.44	32.5	86.7
	-Zn/CK	1.07	0.4	**	0.55	0.09–1.28	53.4	97.7
RDW	-Zn	0.61	0.17	**	0.25	0.1–0.6	43.9	92.2
	CK	0.48	0.34	**	0.31	0.10–0.63	31.7	86.9
	-Zn/CK	1.26	0.5	**	0.72	0.12–1.37	36.4	96
R/S	-Zn	0.26	0.35	**	0.31	0.06–0.58	34.6	91.1
	CK	0.22	0.28	**	0.2	0.13–0.31	17.4	75.3
	-Zn/CK	1.18	1.26	NS	1.19	0.35–1.57	21	88.2

a ** indicates significant differences between Ye478 and Wu312 at P < 0.01, and NS indicates no significant difference between Ye478 and Wu312.

The correlations among different traits were examined and the coefficients of the correlation between each trait pair are shown in Supplementary Tables 9, 10. Spearman’s correlation analysis indicated that the Zn score was positively correlated with the shoot and root dry weights, and was negatively correlated with the R/S ratios (Supplementary Table 9). This was expected because the Zn score was visually scaled by the shoot and root growth in the plants under Zn-deficient conditions (Figure 1). In this study, ZE was mainly characterized by the ratios of the dry weights under -Zn conditions to the values under CK conditions. And these also implied that plants with higher zinc scores also have higher shoot and root dry weights, even higher Zn efficiencies, as well as lower R/S ratios. Additionally, Pearson’s correlation analysis showed that the shoot dry weight displayed a highly positive correlation with the root dry weight at the same Zn nutritional status (*r* > 0.772, *P* < 0.01) (Supplementary Table 10), indicating that plants with higher shoot biomass accumulation may also obtain better root growth. And a positive correlation between two Zn-efficiency indexes was found (*r* = 0.647, *P* < 0.01) (Supplementary Table 10), suggesting that plants with higher ZE based on shoot dry weight also have higher ZE based on root dry weight.

#### Quantitative Trait Locus Detection

**FIGURE 7:**
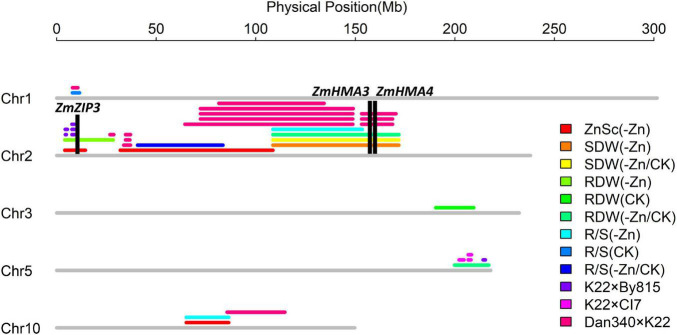
Thirteen loci detected on chromosomes 1, 2, 3, 5, 10 in the Ye478 × Wu312 RIL population and the co-localizations (unpublished data of our lab) identified in the K22 × By815, K22 × CI7, and Dan340 × K22 RIL populations. Important candidate genes (*ZmZIP3*, *ZmHMA3*, *ZmHMA4*) are depicted in black columns.

In this study, thirteen *ZEAL*s were detected using the BLUP for each trait, namely, three for the Zn score, two for the shoot dry weight, and four each for the root dry weight and R/S ratio. These loci were distributed on chromosomes 1, 2, 3, 5, and 10, explaining 7.6–63.5% of the phenotypic variation. The information for each QTL is presented in Table 2, including the peak position, 1-LOD genetic interval and its corresponding physical interval, LOD value, and percentage of total phenotypic variance explained *(R*^2^*)*, additive effects. The physical positions for each locus are displayed in Figure 7.

**TABLE 2:** Quantitative trait loci for traits associated with Zn deficiency tolerance.

**Trait**	**Treatment**	**Name**	**Chr**	**Peak (cM)**	**Marker interval (cM)**	**Physical**	**LOD**	**Add**	***R*^2^ (%)**	**References**
						**Pos (Mb)^a^**		**Effect^b^**		
ZnSc	-Zn	*qZEAL-ZnSc2-1*	2	25.6	umc1542 (9.6)–umc1518 (33.8)	4.1–14.4	19.8	0.75	63.5	
		*qZEAL-ZnSc2-2*	2	70.8	umc2248 (62.8)–umc1003 (84.3)	31.9–108.6	3.9	0.26	10.1	Zhang et al., 2017
		*qZEAL-ZnSc10-1*	10	62.7	umc1345 (44.7)–umc1336 (81.6)	65.1–86.4	15.3	–0.73	54.8	
SDW	-Zn	*qZEAL-SDW2-1*	2	94.3	umc1003 (84.3)–umc1875 (103.3)	108.6–171.8	6.1	0.27	20.7	Li et al., 2016; Zhang et al., 2017; Hindu et al., 2018
	-Zn/CK	*qZEAL-SDW2-2*	2	96.3	umc1003 (84.3)–umc1875 (103.3)	108.6–171.8	6.3	0.16	17.3	Li et al., 2016; Zhang et al., 2017; Hindu et al., 2018
RDW	-Zn	*qZEAL-RDW2-1*	2	33.8	umc1542 (9.6)–bnlg2248 (44.0)	4.1–28.4	3	0.03	7.6	Li et al., 2016
	CK	*qZEAL-RDW3-1*	3	191.8	bnlg197 (193.0)–phi046 (216.8)	190.4–209.4	4.6	0.05	19	
	-Zn/CK	*qZEAL-RDW2-2*	2	98.8	umc1003 (84.3)–nc003 (104.9)	108.6–171.9	3.3	0.13	10.6	Li et al., 2016; Zhang et al., 2017; Hindu et al., 2018
		*qZEAL-RDW5-1*	5	193.5	umc2306 (177.8)–umc2013 (187.5)	199.8–217.0	3.4	–0.15	16.4	
R/S	-Zn	*qZEAL-R/S2-1*	2	96.8	umc1003 (84.3)–umc1065 (96.8)	108.6–153.5	8.9	–0.05	20.7	Li et al., 2016
		*qZEAL-R/S10-1*	10	78.7	umc1345 (44.7)–umc1336 (81.6)	65.1–86.4	4.4	0.05	17.4	Li et al., 2016
	CK	*qZEAL-R/S1-1*	1	31.9	umc1071 (15.4)–umc1222 (29.9)	7.9–11.5	3.1	–0.01	9.2	
	-Zn/CK	*qZEAL-R/S2-2*	2	64.8	phi083 (55.7)–umc1635 (73.6)	40.6–83.5	4.8	–0.19	15.3	Gu et al., 2015; Li et al., 2016

*^a^Physical position refers to the B73 reference sequence version 2.*

*^b^Positive values of additive effect indicate Ye478 alleles are in the direction of increase; negative values indicate Wu312 alleles are in the direction of increase.*

#### Quantitative Trait Locus for Zinc Score

Three QTLs controlling the Zn score were identified on chromosomes 2 and 10. *qZEAL-ZnSc2-1* is a major QTL detected in the interval of umc1542–umc1518, explaining 63.5% of phenotypic variation. A minor-effect QTL *qZEAL-ZnSc2-2* was identified between umc2248 and umc1003, explaining 10.1% of the phenotypic variation. The alleles from Ye478 at these two loci contributed to the increased Zn scores. Another major effect QTL, *qZEAL-ZnSc10-1*, was mapped in the interval of umc1345–umc1336 on chromosome 10, explaining 54.8% of the phenotypic variation. The additive effect at this locus was originated from Wu312.

#### Quantitative Trait Locus for the Shoot and Root Dry Weights

Six loci controlling the shoot and root dry weights were mapped on chromosomes 2, 3, and 5. Two moderate-effect QTLs, *qZEAL-SDW2-1* and *qZEAL-SDW2-2*, were both located in the genomic region of umc1003–umc1875, explaining 20.7 and 17.3% of the phenotypic variation, respectively. Four QTLs controlling the root dry weights were identified on chromosomes 2, 3 and 5, explaining 7.6–19.0% of the phenotypic variations. Two minor-effect QTLs were identified on chromosome 2 and explained 7.6–10.6% of the phenotypic variation, including *qZEAL-RDW2-1* which was mapped at 4.1–28.4 Mb, and *qZEAL-RDW2-2* which was detected at 108.6–171.9 Mb. Two moderate-effect QTLs, *qZEAL-RDW3-1* detected on chromosome 3 and *qZEAL-RDW5-1* on chromosome 5, accounted for 19.0 and 16.4% of the phenotypic variation, respectively. Except for *qZEAL-RDW5-1*, the alleles from Ye478 at the five loci contributed to the enhanced shoot and root biomass accumulation under low Zn stress as well as ZE based on dry weights.

#### Quantitative Trait Locus for Root to Shoot Ratio

Four QTLs controlling the R/S ratio were identified on chromosomes 1, 2, and 10. Under Zn-deficient conditions, the moderate-effect QTL *qZEAL-R/S2-1* was flanked by umc1003–umc1065 on chromosome 2 and explained 20.7% of the phenotypic variation. Another moderate-effect QTL *qZEAL-R/S10-1* was mapped on chromosome 10 and explained 17.4% of the phenotypic variation. In the CK treatment, a minor-effect QTL *qZEAL-R/S1-1* was detected on chromosome 1, accounting for 9.2% of the phenotypic variation. In the -Zn/CK treatment, a moderate-effect QTL *qZEAL-R/S2-2* (*R*^2^ = 15.3%) was flanked by phi083–umc1635 on chromosome 2.

### Quantitative Trait Locus Co-localization and Candidate Gene Identification

In the Ye478 × Wu312 RIL population, four QTL co-localizations were identified by ten QTLs for different traits. The LOD score profile for each QTL within the four QTL co-localizations is shown in Supplementary Figure 3. The major QTL *qZEAL-ZnSc2-1* (*R*^2^ = 63.5%) was co-localized with the minor-effect QTL *qZEAL-RDW2-1* on chromosome 2 (Supplementary Figure 3A). Additionally, *qZEAL-ZnSc2-1* was co-localized with *qZEAL-R/S2-2* on chromosome 2 (Supplementary Figure 3B). And on chromosome 10, a major-effect QTL *qZEAL-ZnSc10-1* (*R*^2^ = 54.8%) was co-localized with a moderate-effect QTL *qZEAL-R/S10-1* (Supplementary Figure 3D). The significant correlation (*P* < 0.01) between the Zn score and the shoot and root dry weight and the R/S ratio in the -Zn and -Zn/CK treatments, implied that these two major-effect QTL controlling Zn score may have pleiotropic effects on the biomass accumulation and their allocation in shoots and roots at low Zn nutritional status. In addition, two QTLs controlling the shoot dry weight (*qZEAL-SDW2-1 qZEAL-SDW2-2*) were co-localized with *qZEAL-RDW2-2* and *qZEAL-R/S2-1* which controlled the root dry weight and R/S ratio, respectively (Supplementary Figure 3C). There were strong positive correlations (*P* < 0.01) among the shoot and root dry weights and the R/S ratio in the -Zn and -Zn/CK treatments. These indicated that QTLs for the shoot dry weight may also affect the root growth and both of them may share the same genetic base in response to Zn deficiency stress.

Additionally, based on physical position, eleven genomic regions were identified in the QTL co-localization detected by the QTLs in this study and the QTLs in other three different RIL populations (K22 × By815, K22 × CI7, Dan340 × K22, unpublished data). As shown in Figure 7, on chromosome 2, the major QTL *qZEAL-ZnSc2-1* and minor-effect QTL *qZEAL-RDW2-1* were co-localized with three QTLs mapped in the K22 × By815 (KB) population which accounted for 9.2–12.0% of the phenotypic variation. Two intervals were identified at 4.4–4.9 and 7.7–8.8 Mb. Additional two intervals (26.8–28.7, 33.7–37.1 Mb) on chromosome 2 were narrowed by three loci which explained 9.3–13.1% of the phenotypic variation in the Dan340 × K22 (DK) population. At 108.6–134.3 Mb, the moderate-effect QTL *qZEAL-SDW2-1* (*R*^2^ = 20.7%), together with *qZEAL-SDW2-2*, *qZEAL-RDW2-2*, and *qZEAL-R/S2-1* were co-localized with four QTLs which accounted for 11.9–23.3% of the phenotypic variance in the DK and KB population. At 153.1–168.7 Mb, *qZEAL-SDW2-1*, *qZEAL-RDW2-2*, and *qZEAL-SDW2-2* were co-localized with the locus which explained 21.2% of the phenotypic variation in the DK population. On chromosome 5, three genetic intervals (202.1–204.6, 206.6–208.1, 214.1–215.3 Mb) were located in the QTL co-localization of *qZEAL-RDW5-1* with four loci detected in the KB and DK population (Figure 7). On chromosome 10, the moderate-effect QTL *qZEAL-R/S10-1* (*R*^2^ = 17.4%) was co-localized with the major-effect QTL *qZEAL-ZnSc10-1* (*R*^2^ = 54.8%) in this study.

These genomic regions reduced by the co-localization identified on chromosomes 2, 5, and 10 were selected for candidate genes identification (Supplementary Table 12). In total, 601 candidate genes were identified within the eleven intervals reduced by the QTL co-localization: 317 genes on chromosome 2, 145 genes on chromosome 5, 139 genes on chromosome 10 (Supplementary Table 11). Among them, eight candidate genes were considered to be associated with Zn efficiency in maize, including *ZmZIP3*, *ZmHMA3*, and *ZmHMA4* (Table 3).

**TABLE 3:** The information of candidate genes associated with Zn deficiency tolerance.

**Chr**	**Gene ID**	**Description**
2	GRMZM2G045849	ZmZIP3—Zinc-regulated, iron-regulated transporter-like protein 3
2	GRMZM2G021849	UDP-Glycosyltransferase superfamily protein
	GRMZM2G009626	UDP-Glycosyltransferase superfamily protein
	GRMZM2G463996	UDP-Glycosyltransferase superfamily protein
	GRMZM2G175576	ZmHMA3—Cadmium/Zinc-transporting ATPase 3
	GRMZM2G455491	ZmHMA4—Cadmium/Zinc-transporting ATPase 4
5	GRMZM2G030465	ZmIAA17—Aux/IAA-transcription factor 17
10	GRMZM2G145870	ZmIGPS—Indole-3-glycerol phosphate synthase

### Expression of *ZIP* Genes in Maize

The results of Experiment 1 have shown that the differential ZEs of the most tolerant (Ye478) and the most sensitive (Wu312) inbred lines were mainly due to their differences in Zn uptake and transport efficiency. In addition, *ZmZIP3*, a member of the *ZIP* gene family which encodes major Zn transporters in maize, was identified in the major QTL *qZEAL-ZnSc2-1* (*R*^2^ = 63.5%) in the Ye478 × Wu312 RIL population. Therefore, based on Experiments 1 and 2 (Figures 4A,B), Experiment 3 was designed to analyze the expression of eight *ZIP* genes (*ZmZIP1*-*ZmZIP8*) under -Zn and CK conditions (Figure 4C).

**FIGURE 8:**
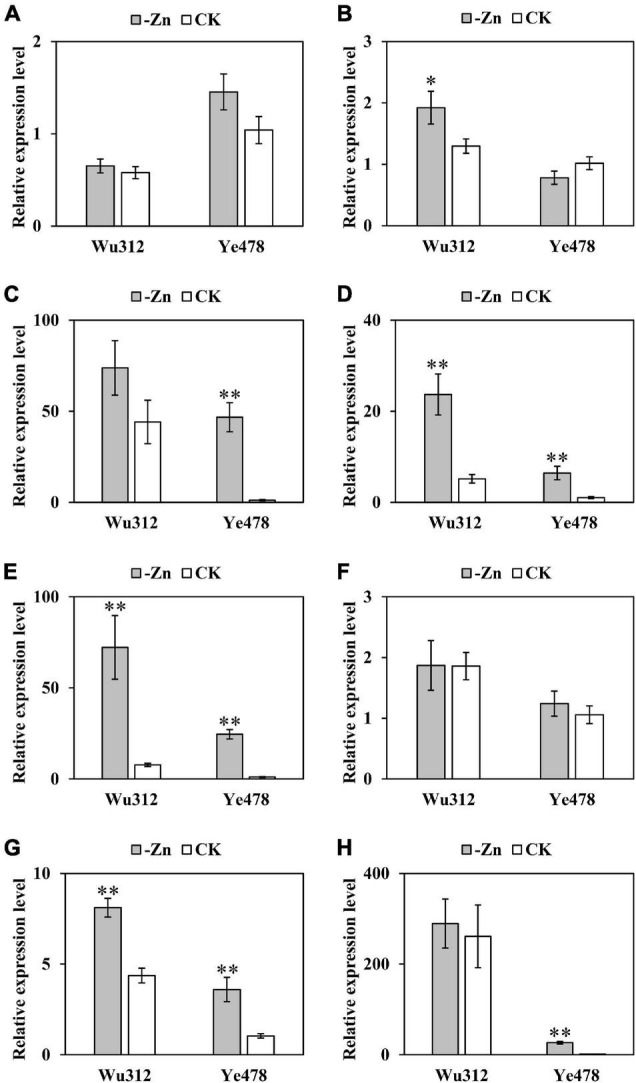
Expression of eight *ZIP* genes in maize. Relative expression levels of eight *ZmZIPs* (*ZmZIP1- ZmZIP8*) in the roots of Wu312 and Ye478 grown hydroponically in a mixed-crop system for 21 days after transplanting in the -Zn (0.3 μmol L^–1^ Zn-EDTA) and CK (10 μmol L^–1^ Zn-EDTA) treatment. **(A–H)** show the relative expression levels of *ZmZIP1- ZmZIP8*, respectively. ^∗^ and ^∗∗^ indicate the significant differences between the -Zn and CK treatments at *P* < 0.05 and *P* < 0.01, respectively.

The expression of *ZmZIP 1*-*8* under -Zn and CK conditions was shown in Figure 8. *ZmZIP3*, *ZmZIP4*, *ZmZIP5*, *ZmZIP7*, and *ZmZIP8* in the roots of Ye478 showed at least 39-, 6-, 22-, 3-, and 24-fold upregulation in response to Zn deficiency, respectively. Zn deficiency had no significant effects on the expressions of *ZmZIP1*, *ZmZIP2*, and *ZmZIP6* in the roots of Ye478. The relative expression level of *ZmZIP2*, *ZmZIP4*, *ZmZIP5*, and *ZmZIP7* in the Zn-deficient roots of Wu312 were increased by 1. 5-, 4. 5-, 9. 3-, and 1.9-fold, respectively. The other four genes (*ZmZIP1*, *ZmZIP3*, *ZmZIP6*, and *ZmZIP8*) were not upregulated in response to Zn deficiency. Notably, *ZmZIP3* and *ZmZIP8* were barely expressed in the roots of Ye478 under CK conditions but showed high gene expression under Zn deficiency. By contrast, there was no significant difference in the expression level of these two genes in the roots of Wu312 between -Zn and CK conditions. Additionally, the upregulations of *ZmZIP4*, *ZmZIP5*, and *ZmZIP7* were higher in the Zn-deficient roots of the tolerant line (Ye478) than in those of the sensitive line (Wu312). These facts indicated that the five *ZIP* genes (*ZmZIP3*, *ZmZIP4*, *ZmZIP5*, *ZmZIP7*, and *ZmZIP8*) may be important for Zn deficiency tolerance variations in maize.

## Discussion

### Physiological Mechanisms Underlying Zn Efficiency

Root to shoot ratio increase, an initial response to Zn deficiency, is also one of the most sensitive indicators to characterize ZE. This response indicates a compensation mechanism that plants could allocate the photosynthetic products produced by the shoot to root growth (Rengel and Graham, 1995), which may lead to greater morphological changes in the roots (Genc et al., 2007). Besides, the PCA analysis showed that Ye478 had a higher Zn uptake, transport efficiency, as well as lower R/S ratio efficiency, compared with Wu312 (Figure 5), indicating that the molecular mechanisms underlying Zn uptake and transport may be vital in explaining the differences in ZE between Ye478 and Wu312.

The Zn-efficient genotypes may not necessarily have higher Zn concentration in the tissue or grain (Graham et al., 1992; Cakmak et al., 1996). Our results further confirmed that the Zn concentrations in the shoots and roots were not correlated with ZE (Figure 3) and were not suitable to characterize ZE in maize at the seedling stage. The shoot Zn concentrations of the Zn-efficient inbred lines (Ye478, CI7) were not significantly higher than the Zn-inefficient inbred lines (Wu312, K22), mainly due to the dilute effect (Marschner, 1995). The Zn-efficient inbred lines, which do not maintain higher shoot Zn concentrations, can still produce more biomass than the Zn-inefficient inbred lines, possibly attributed to the advantages in the subcellular Zn compartmentation and homeostasis (Outten and O’Halloran, 2001; Hacisalihoglu et al., 2004) and the more efficient biochemical utilization of the cellular Zn (Cakmak, 2000; Hacisalihoglu et al., 2003). The Zn concentrations in the shoots and roots declined significantly under Zn-deficient conditions, but the Fe and Cu concentrations in the shoots and the Mn concentrations in the roots increased obviously. This may be due to the competitive interaction of the Zn deficiency-inducible transport proteins in the transport of Fe, Mn, Cu compared with Zn across the plasma membrane (Rengel and Graham, 1996; Tiong et al., 2015).

### Comparison of Detected Quantitative Trait Locus With Previous Reports

There were few reports on the QTL mapping of Zn deficiency tolerance in maize and only a small number of researches related to Zn nutrition in maize, which were mostly concentrated on Zn and other mineral concentration or content in grain, have been reported (Šimić et al., 2012; Gu et al., 2015; Jin et al., 2015; Zhang et al., 2017; Hindu et al., 2018; Ju et al., 2018). On chromosome 2, five QTLs (*qZEAL-ZnSc2-2*, *qZEAL-SDW2-1*, *qZEAL-SDW2-2*, *qZEAL-RDW2-2*, *qZEAL-R/S2-2*) detected in our work were co-localized with the loci controlling kernel mineral concentration in maize (Table 2). Among these five QTLs, the overlapped genomic regions of *qZEAL-SDW2-1*, *qZEAL-SDW2-2*, and *qZEAL-RDW2-2*, were co-localized with the QTLs controlling Fe concentration in grain by linkage analysis, and the SNP associated with kernel Fe concentration by GWAS (Zhang et al., 2017; Hindu et al., 2018). Additionally, *qZEAL-ZnSc2-2* and *qZEAL-R/S2-2* were co-localized with the QTL for the grain Fe concentration and the QTL controlling the kernel Mn concentration, respectively (Gu et al., 2015; Zhang et al., 2017). Apart from that, on chromosomes 2 and 10, seven QTLs detected in the -Zn and -Zn/CK treatment of this study, were co-localized with the loci associated with the root dry weight and root morphological traits under high- and low-nitrogen levels (Li et al., 2016). These results implicate that these QTL regions may have pleiotropic effects on the mineral concentration of grains and root traits in maize. Overall, 62% of the QTLs have been identified to be co-localized by the loci detected in the previous studies (Table 2), suggesting that these QTLs detected by different traits may be highly reliable for candidate gene identification.

### Expression Patterns of *ZIP*s in Maize

Despite *ZIPs* having been characterized in many plants, there are little researches concerning the expressions of *ZIP*s in maize. The expression patterns for *ZmZIPs* in our study showed that *ZmZIP4*, *ZmZIP5* and *ZmZIP7* were induced in Zn-deficient roots, which is consistent with Li et al. (2013) and Mager et al. (2018), indicating that *ZmZIP4*, *ZmZIP5*, *ZmZIP7*, and *ZmZIP8* may play important roles in the uptake and transport of Zn. The *ZIP*s from maize and rice may share a common evolutionary ancestor, and they existed as orthologs, including *ZmZIP3* and *OsZIP3*, *ZmZIP4* and *OsZIP4*, *ZmZIP5*, *ZmZIP7* and *OsZIP7*, and *ZmZIP8* and *OsZIP8* (Li et al., 2013). In rice, *OsZIP4*, *OsZIP7*, and *OsZIP8* are strongly induced in Zn-deficient shoots and roots and complement the growth defect of the Zn-uptake yeast mutant, indicating that they are functional transporters of Zn (Ishimaru et al., 2005; Lee et al., 2010a; Kabir et al., 2017). *OsZIP4*, an ortholog of *ZmZIP4*, which is mainly expressed in the vascular bundles of Zn-deficient roots (Lee et al., 2010b) and meristem of Zn-deficient roots and shoots (Ishimaru et al., 2005), may be responsible for regulating Zn supply in developing young leaves and transporting Zn over a long distance from the old to young leaves (Lee et al., 2010b). *OsZIP7* which is the ortholog for both *ZmZIP5* and *ZmZIP7* regulates the distribution of Zn within rice, including xylem loading into roots and inter-vascular transfer in nodes, to preferentially deliver Zn to developing tissues (Tan et al., 2019).

### Candidate Genes Associated With Zinc Deficiency Tolerance

*ZmZIP3*, also known as *GRMZM2G045849*, was detected in the major QTL *qZEAL-ZnSc2-1* which explained 63.5% of the phenotypic variation. It was reported that *ZmZIP3* is induced in Zn-deficient roots and efficiently reverses the growth of yeast mutant defectives in Zn uptake caused by Zn deficiency (Li et al., 2013). Notably, distinct expression patterns of *ZIP* genes exist between Zn tolerant and sensitive genotypes under Zn deficiency (Khatun et al., 2018). In contrast to the mild upregulation of *ZmZIP3* in Zn deficiency recorded by Mager et al. (2018), there was 39-fold upregulation of *ZmZIP3* in the Zn-deficient roots of Ye478, whereas there was no significant difference of expression level in Wu312 between -Zn and CK conditions. This indicated that *ZmZIP3* may be associated with the ZE difference between Zn-efficient and Zn-inefficient inbred lines.

*OsZIP3*, an ortholog of *ZmZIP3*, was detected in the vascular bundles and epidermal cells inside the stem in rice (Ramesh et al., 2003). More recently, OsZIP3 has been found to be located at the xylem, intervening the parenchyma cells and xylem transfer cell of the enlarged vascular bundle (EVB) in the nodes, and show a high expression in the nodes (Sasaki et al., 2015). The experiment with the Zn stable isotope and knockdown analysis confirmed that OsZIP3 is required for unloading Zn from the xylem of EVB, which initiates a preferential distribution to the developing tissues in the shoot (Sasaki et al., 2015). HvZIP3, which is 83% identical with OsZIP3, restores the growth of the yeast mutant defective in Zn^2+^ uptake grown under Zn^2+^-depleted conditions (Pedas et al., 2009). *HvZIP3*, an ortholog of *ZmZIP3*, is highly and consistently induced in Zn-deficient roots and plays a role in the uptake and root-to-shoot translocation of Zn under Zn deficiency, which is supported by the results of the tissue-specific expression in the roots (Tiong et al., 2015). Hence, the *ZmZIP3* mapped in this research, which is the ortholog for *OsZIP3* and *HvZIP3*, is suggested to play an essential role in Zn uptake and transport in maize.

In this research, *ZmHAM3* and *ZmHMA4* (*GRMZM2G175576*, *GRMZM2G455491*) encoding Zn transporters were identified in the co-localizations on chromosome 2. *AtHMA2* which is the homologous gene of *ZmHMA3* has N- and C-terminal domains that could bind Zn^2+^ with high affinity. *AtHMA2* is expressed in the root vasculature and is responsible for the root-to-shoot translocation of Zn via xylem loading (Eren and Arguello, 2004; Wong et al., 2009). Hussain et al. (2004) have done research on *hma2, hma4* double mutants, and found that *hma2*, *hma4* double mutants have a severe shoot Zn-deficiency phenotype and accumulate Zn in roots. The results also showed that the levels of Zn, but not other essential elements, in the shoot tissues of a *hma2*, *hma4* double mutant are decreased compared with the wild type.

*OsHMA3*, the homologous gene of *ZmHMA3* in rice, is the first QTL for cadmium (Cd) accumulation in rice which has been cloned by forwarding genetics (Ueno et al., 2010; Liu et al., 2019). *OsHMA3* is preferentially expressed in the roots and is involved in the sequestration of Cd into the vacuoles of the roots. Some rice cultivars possess weak or loss-of-function alleles of *OsHMA3* and the difference in the messenger RNA (mRNA) expression level of *OsHMA3*, accumulating high levels of Cd in the shoots and grain (Miyadate et al., 2011; Yan et al., 2016; Cai et al., 2019). When *OsHMA3* is overexpressed under the control of the ubiquitin or *OsHMA2* promoter, not only Cd but also Zn in the roots and the root cell saps are increased (Shao et al., 2018). Several genes related to Zn transporters are upregulated in the *OsHMA3*-overexpressed lines (Sasaki et al., 2014). Zn sequestered by *OsHMA3* in the roots provides an important source for the shoot under conditions of Zn deficiency (Yamaji et al., 2013; Sasaki et al., 2015). Up to now, many *HMA* genes have been identified and studied in *Arabidopsis* and rice. However, the analysis of the *HMA* gene family in maize is still scarce. Further characterization of these transporters is crucial to understand the mechanism underlying the uptake and transport of Zn in maize.

Under abiotic stress, plants scavenge reactive oxygen species (ROS) to restore redox metabolism and to keep cellular turgor and structures actively functioning (Yancey, 2005; Mittler, 2006). Zn deficiency causes the accumulation of ROS, resulting in the oxidative degradation of the indole-3-acetic acid (IAA) and repression in the shoot growth (Marschner, 1995). Furthermore, Zn is involved in the detoxification of superoxide radicals and the synthesis of phytohormones (Cakmak, 2000), directly participating in the synthesis of IAA (Alloway, 2008). Variations in hormones may be signals in response to nutrient stress, resulting in morphological and physiological changes in plants. It is reported that the IAA level of the shoot tips and young leaves in -Zn plants is about 50% lower than that in CK plants (Cakmak et al., 1989). The genes associated with the signaling and metabolism of IAA may be related to the mechanisms in tolerating Zn-deficiency stress.

To prevent auxin-responsive transcription in the path from the auxin signal perception to the altered gene expression, Aux/IAA proteins generally function as repressors (Korasick et al., 2014). This pathway requires three components: auxin-perceiving TIR1/AFB F-box proteins, Aux/IAA repressor proteins, and auxin response factors (ARF) transcription factors (Weijers and Wagner, 2016). When the auxin level is higher, ARFs are released from the inhibition of Aux/IAAs, which allows ARF proteins to reach their AuxRE targets to derepress/activate the early auxin response genes (such as *GH3* and *SAUR* gene family). When the auxin concentration decreases below a threshold, the Aux/IAAs are associated with ARFs and those auxin-response genes are repressed (Roosjen et al., 2018; Figure 4D).

In maize, ZmAFB2 shared high amino acid sequence homology with *Arabidopsis* AtAFB2 and AtAFB3 and contained one F-Box region and four LRR regions (Yang et al., 2013). It has been reported that *miR393* targets several auxin receptors for degradation such as *TIR1*, *AFB2*, and *AFB3* (Navarro et al., 2006). The *ZmAFB2* contained the *miR393* cleaved site. Iglesias et al. (2011) demonstrated that salicylic acid (SA) might inhibit auxin signaling by the translational repression of TIR1/AFB proteins during stress. *ZmAFB2* may be involved in the crosstalk between SA and auxin signal transduction (Yang et al., 2013). The auxin-responsive gene *Zm00001d045203*, which encodes an Aux/IAA inhibitor involved in the SCF^TIR1/AFB^-mediated auxin signaling pathway, is the homolog of *Arabidopsis thaliana IAA29* (Zhang et al., 2019).

Among the 34 members of the maize *Aux/IAA* gene family, all but one (*ZmIAA23*) tested maize *Aux/IAA* genes were auxin-inducible, displaying two types of auxin induction within 3 h of treatment (Ludwig et al., 2013). Putative *cis*-acting regulatory DNA elements involved in auxin response, light signaling transduction, and abiotic stress adaption (such as dehydration-responsive element, low temperature-responsive element), were observed in the promoters of *ZmIAA* genes (Wang et al., 2010). The diversity of the *cis*-elements in the promoters of *ZmIAA* genes is related to the key role of Aux/IAA proteins in plant life. The maize *lateral root primordia 1* (*lrp1*) encodes a transcriptional activator that is directly regulated by the Aux/IAA protein ROOTLESS WITH UNDETECTABLE MERISTEM 1 (RUM1 corresponding to ZmIAA10), which regulates the initiation of lateral and seminal root (von Behrens et al., 2011; Zhang et al., 2014; Wang et al., 2021). RUM1 acts as a transcriptional repressor interacting with ZmARF25 or ZmARF34, thereby regulating the transcription of auxin-responsive genes in pericycle cells of primary roots (Zhang et al., 2015).

An analysis of the conserved motifs indicated that 23 of 35 ZmARF proteins contain domains III and IV, which were also found in the C-terminus of Aux/IAAs (Liu Y. et al., 2011; Saidi and Hajibarat, 2020). These domains have been shown to mediate both homo- and hetero-dimerization between the members of the Aux/IAA and ARF families (Kim et al., 1997; Galli et al., 2018). Twelve maize ARFs with glutamine-rich middle regions could be activators in modulating the expression of auxin-responsive genes (Wang et al., 2012). The expression of *ARF* genes in maize is regulated by auxin and small RNAs. The dynamic expression patterns of *ZmARF* genes were observed in different stages of embryo development (Xing et al., 2011).

The GRMZM2G030465 on chromosome 5, also known as *ZmIAA17* which is a member of the *Aux/IAA* gene family (Ludwig et al., 2013), was detected at the locus *qZEAL-RDW5-1*. *AtIAA13*, an orthologous of *ZmIAA17* in *Arabidopsis*, is highly expressed in the cortex and lateral root primordia, suggesting that *AtIAA13* functions in initiating the formation of the lateral roots (Yamauchi et al., 2019). *OsIAA9*, an ortholog of *ZmIAA17* in rice, is also greatly induced by multiple hormones and abiotic stresses (Luo et al., 2015). The ectopic overexpression of *OsIAA9* results in fewer crown and lateral roots and reduced the inhibition of root elongation by auxin, suggesting that OsIAA9 is a negative regulator of auxin-regulated root growth (Song, 2019). Additionally, *OsIAA9* is confirmed to regulate gravitropic response by controlling granules accumulation and distribution in root tips (Luo et al., 2015).

*GRMZM2G021849*, *GRMZM2G009626*, and *GRMZM2G463996* on chromosome 2, co-localized by four QTLs in the Ye478 × Wu312 and Dan340 × K22 population, were identified to be members of UDP-Glycosyltransferase superfamily, which are likely associated with IAA inactivation (Tanaka et al., 2014). Some UDP-glycosyltransferases (UGTs) catalyze the glucosylation of plant hormones, including auxin, ABA, cytokinins, and SA using UDP-glucose as a co-substrate (Lim and Bowles, 2004; Gachon et al., 2005). It was reported that *UGT84B1*, *UGT74E2*, and *UGT74B1* catalyzed the conversion of IAA to IAA-Glc (Jackson et al., 2001; Grubb et al., 2004; Tognetti et al., 2010). Furthermore, *GRMZM2G145870*, also known as indole-3-glycerol phosphate synthase (*ZmIGPS*) in maize, was identified within *qZEAL-ZnSc10-1*, which was the second largest effect QTL. Indole-3-glycerol phosphate synthase is the only known enzyme to catalyze the formation of the indole ring, which is the key for the tryptophan-independent IAA synthesis pathway in plants (Normanly and Bartel, 1999; Ouyang et al., 2000). Taken together, the members of the UGT family, IAA9, and IGPS3 may be involve in the biosynthesis of IAA and likely affect plant growth and development. Nevertheless, specific functions for these candidate genes in maize are still lacking, and further systematic studies are required.

In summary, there are few systematic studies and explicit demonstrations of the molecular mechanism underlying the tolerance of Zn deficiency stress. In our study, members of the *ZIP* gene family and *HMA* gene family were detected by linkage analysis. ZIP3 is required for unloading Zn from the xylem of enlarged vascular bundles and for the preferential distribution to the developing tissues in the shoot (Sasaki et al., 2015). HMA3 is localized to the tonoplast of all root cells (Ueno et al., 2010) and sequester Zn^2 +^ into the vacuole (Cai et al., 2019). HMA4 plays an essential role in the root-to-shoot translocation of Zn and as an efflux pump (Wong and Cobbett, 2009). Additionally, Zn deficiency causes ROS in plants, which leads to fluctuating auxin levels. In the auxin-dependent gene regulation pathway, Aux/IAA acts as a repressor of auxin transcription factors (Weijers and Wagner, 2016). Under low-auxin levels, Aux/IAA is bond to ARF functioning as a repressor to prevent transcription. Under high-auxin levels, Aux/IAA disassociates ARF and is bonded to TIR1/AFB with auxin, then ARF is released from the inhibition of Aux/IAA and derepresses/activates the regulation of downstream genes (Roosjen et al., 2018; Figure 4D). These genes mentioned above may play very important roles in the mechanism of Zn deficiency stress tolerance.

## Data Availability Statement

The original contributions presented in the study are included in the article/Supplementary Material, further inquiries can be directed to the corresponding author/s.

## Author Contributions

JX and XW performed the experiments and analyzed the data. JX, XW, and HZ wrote the manuscript. FY designed the study and modified the manuscript. All authors contributed to the article and approved the submitted version.

## Conflict of Interest

The authors declare that the research was conducted in the absence of any commercial or financial relationships that could be construed as a potential conflict of interest.

## Publisher’s Note

All claims expressed in this article are solely those of the authors and do not necessarily represent those of their affiliated organizations, or those of the publisher, the editors and the reviewers. Any product that may be evaluated in this article, or claim that may be made by its manufacturer, is not guaranteed or endorsed by the publisher.

## Footnotes

1
http://www.maizeGDB.org
