# Supplementary material for: Identification and Analysis of Zinc Efficiency-Associated Loci in Maize
Source: Front Plant Sci. 2021 Nov 15;12:739282. doi: 10.3389/fpls.2021.739282 (PMC8634756; doi:10.3389/fpls.2021.739282)
Supplement: Supplementary file 2 [file Data_Sheet_1.docx]

**Supplementary Material S1 (Figure S1-S3, Table S1-S11)**

**Identification and Analysis of Zinc Efficiency-Associated Loci in Maize**

Jianqin Xu^1^, Xuejie Wang^1^, Huaqing Zhu^1^, Futong Yu^1†^

^1^ Key Laboratory of Plant-Soil Interaction (MOE), Centre for Resources, Environment and Food Security, College of Resources and Environmental Sciences, China Agricultural University, Beijing 100193, China

† Futong Yu (Corresponding Author): futongyu@cau.edu.cn

Other e-mail addresses:

Jianqin Xu: xujqcau@163.com

Xuejie Wang: wangxjcau@163.com

Huaqing Zhu: zhuhqcau@163.com


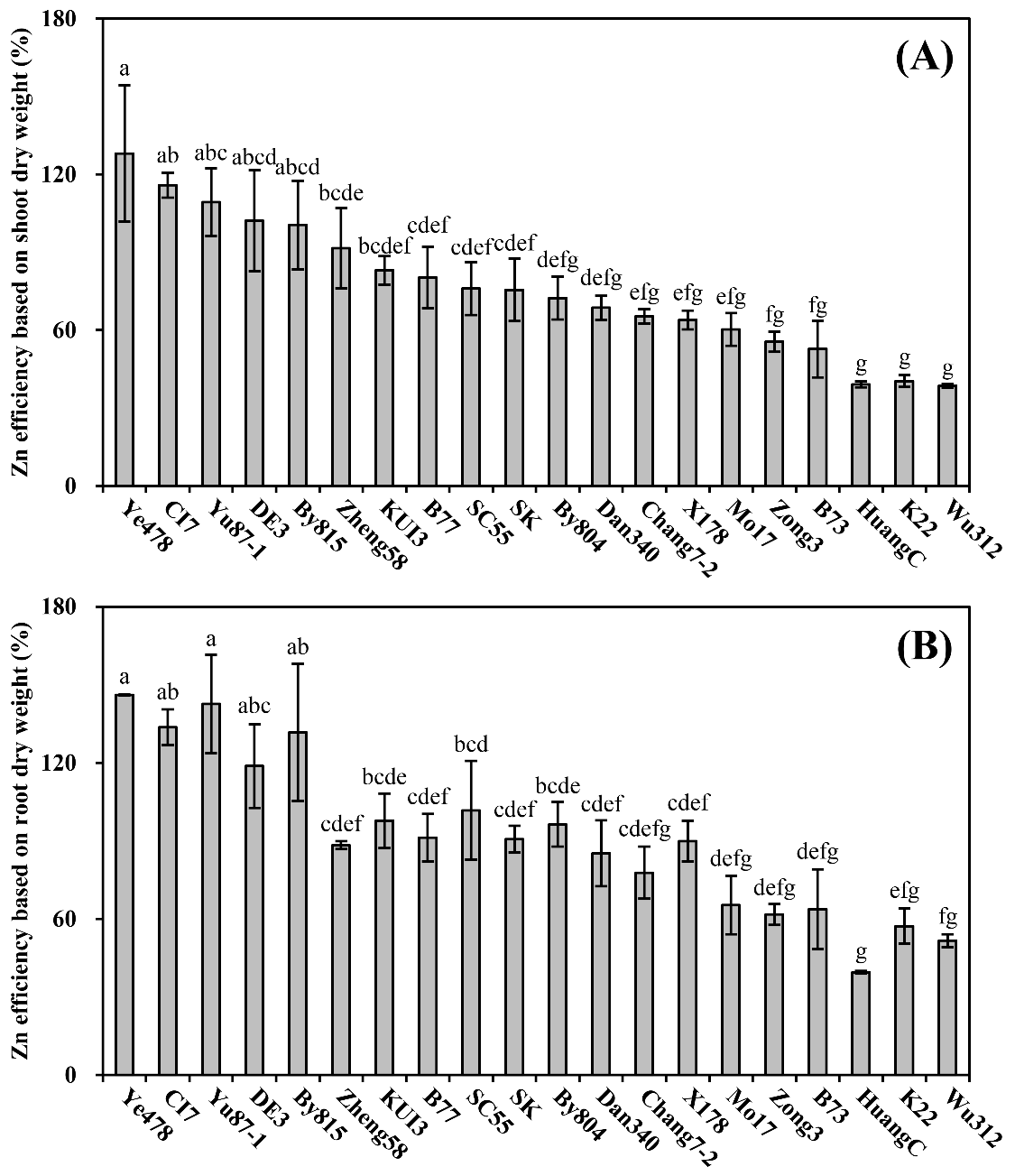


**FIGURE S1** Zn efficiencies (%) based on shoot **(A)** and root **(B)** dry weights of twenty maize inbred lines grown in hydroponics for 21 days after transplanting. Different letters indicate significant differences (*P* < 0.05) among twenty maize inbred lines.


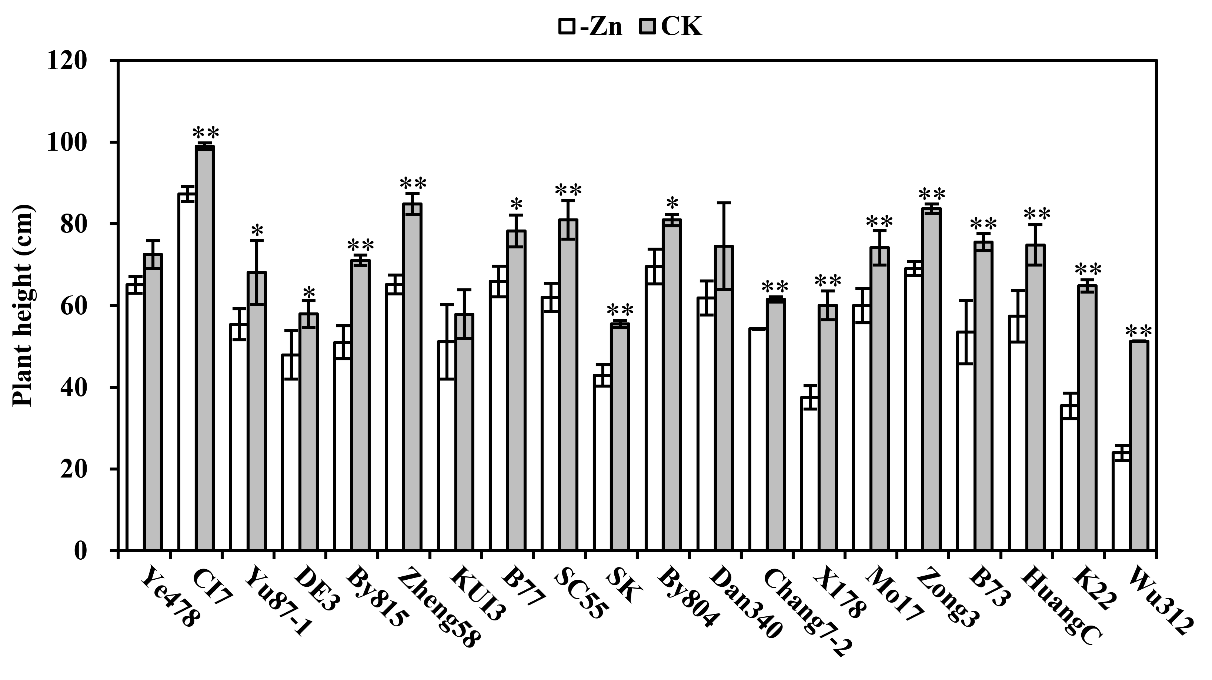


**FIGURE S2** Plant heights of twenty maize inbred lines grown hydroponically in a mixed-crop system for 21 days after transplanting in the -Zn (0.3 μmol L^-1^ Zn-EDTA) and CK (10 μmol L^-1^ Zn-EDTA) treatments. * and ** indicate significant differences between the -Zn and CK treatments at *P* ˂ 0.05 and *P* ˂ 0.01, respectively.


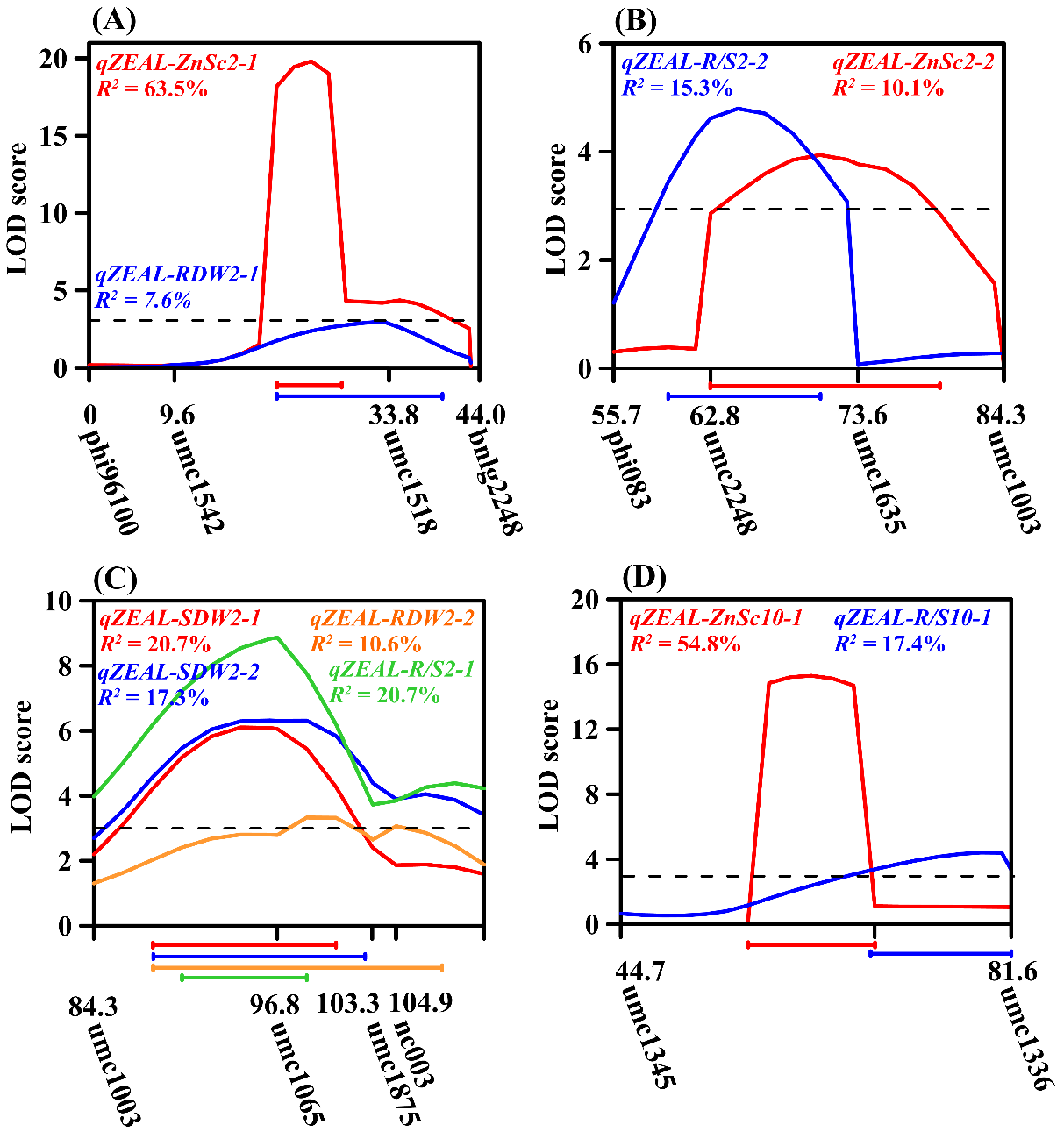


**FIGURE S3** LOD score profiles for the QTLs within four QTL co-localizations in the Ye478 × Wu312 RIL population. Four co-localizations were detected by *qZEAL-ZnSc2-1* and *qZEAL-RDW2-1* **(A)**, *qZEAL-ZnSc2-2* and *qZEAL-R/S2-2* **(B)**, *qZEAL-SDW2-1*, *qZEAL-SDW2-2*, *qZEAL-RDW2-2* and *qZEAL-R/S2-1* **(C)**, *qZEAL-ZnSc10-1* and *qZEAL-R/S10-1* **(D)**. The markers and their genetic positions (cM) were marked at the bottom of the LOD curve for each QTL. Dash line indicates LOD threshold for QTL detection. Solid line (red, blue, orange or green) below the X-axis represents the confidence interval for each QTL using 1-LOD interval method.

| **TABLE S1** Shoot and root dry weights, ratios R/S and relative R/S ratio efficiency (%) in the -Zn and CK treatments | | | | | | | | | | | | | |
| --- | --- | --- | --- | --- | --- | --- | --- | --- | --- | --- | --- | --- | --- |
| Inbred  lines | Shoot dry weight (g plant^-1^) | | |  | Root dry weight (g plant^-1^) | | |  | R/S ratio | | |  | Relative R/S ratio |
|  | -Zn ^a^ | CK | *P* ^b^ |  | -Zn | CK | *P* |  | -Zn | CK | *P* |  | efficiency (%) |
| Ye478 | 3.82 a | 3.05 abcd | 0.128 |  | 0.98 b | 0.67 bcd | 0.015 |  | 0.26 de | 0.22 bcd | 0.174 |  | 115.7 abc |
| CI7 | 4.47 a | 3.86 ab | 0.050 |  | 1.31 a | 0.99 ab | 0.048 |  | 0.29 cd | 0.25 abc | 0.114 |  | 115.7 abc |
| Yu87-1 | 1.94 bcde | 1.86 cdefg | 0.450 |  | 0.58 defg | 0.45 cde | 0.162 |  | 0.30 cd | 0.25 abc | 0.100 |  | 125.0 abc |
| DE3 | 1.15 ef | 1.02 fg | 0.256 |  | 0.35 fgh | 0.27 de | 0.100 |  | 0.31 cd | 0.26 abc | 0.002 |  | 117.5 abc |
| By815 | 2.42 bc | 2.43 cde | 0.489 |  | 1.00 b | 0.79 abc | 0.116 |  | 0.42 ab | 0.32 a | 0.037 |  | 131.0 ab |
| Zheng58 | 2.48 bc | 2.69 bcde | 0.339 |  | 0.78 bcd | 0.67 bcd | 0.282 |  | 0.32 cd | 0.25 abc | 0.001 |  | 129.7 ab |
| KUI3 | 1.53 cdef | 1.93 cdefg | 0.025 |  | 0.43 efgh | 0.45 cde | 0.279 |  | 0.28 cde | 0.22 bcd | 0.006 |  | 125.4 abc |
| B77 | 2.51 bc | 3.12 abc | 0.076 |  | 0.74 bcde | 0.81 abc | 0.162 |  | 0.29 cd | 0.26 abc | 0.034 |  | 114.0 abc |
| SC55 | 1.84 bcde | 2.45 cde | 0.121 |  | 0.51 defgh | 0.51 cde | 0.484 |  | 0.28 cde | 0.21 cd | 0.001 |  | 133.0 ab |
| SK | 0.60 f | 0.79 g | 0.015 |  | 0.19 h | 0.21 e | 0.206 |  | 0.31 cd | 0.26 abc | 0.018 |  | 121.2 abc |
| By804 | 2.63 b | 3.83 ab | 0.037 |  | 0.93 bc | 1.03 ab | 0.251 |  | 0.35 bc | 0.27 abc | 0.001 |  | 133.3 ab |
| Dan340 | 1.55 cdef | 2.25 cdef | 0.084 |  | 0.48 defgh | 0.56 cde | 0.285 |  | 0.33 cd | 0.25 abc | 0.015 |  | 132.9 ab |
| Chang7-2 | 1.27 ef | 1.94 cdefg | 0.002 |  | 0.25 gh | 0.31 de | 0.099 |  | 0.20 e | 0.16 d | 0.048 |  | 118.2 abc |
| X178 | 1.09 ef | 1.70 defg | 0.009 |  | 0.48 defgh | 0.54 cde | 0.160 |  | 0.44 a | 0.32 a | 0.001 |  | 140.5 ab |
| Mo17 | 0.95 ef | 1.59 efg | 0.020 |  | 0.30 gh | 0.47 cde | 0.080 |  | 0.31 cd | 0.29 ab | 0.276 |  | 108.6 bc |
| Zong3 | 2.29 bcd | 4.11 a | 0.001 |  | 0.66 cdef | 1.07 a | 0.012 |  | 0.29 cd | 0.26 abc | 0.078 |  | 111.1 abc |
| B73 | 1.38 def | 2.34 cdef | 0.051 |  | 0.36 fgh | 0.51 cde | 0.112 |  | 0.26 de | 0.22 bcd | 0.003 |  | 130.6 ab |
| HuangC | 0.78 f | 1.99 cdefg | 0.003 |  | 0.20 h | 0.51 cde | 0.005 |  | 0.26 de | 0.25 abc | 0.201 |  | 95.4 c |
| K22 | 0.74 f | 1.85 cdefg | 0.003 |  | 0.26 gh | 0.47 cde | 0.011 |  | 0.36 bc | 0.25 abc | 0.001 |  | 141.4 a |
| Wu312 | 0.69 f | 1.79 cdefg | 0.003 |  | 0.24 gh | 0.48 cde | 0.012 |  | 0.36 bc | 0.27 abc | 0.098 |  | 133.9 ab |
| a Different letters indicate significant differences among twenty maize inbred lines in the -Zn or CK treatment at *P* ˂ 0.05.  b *P* ˂ 0.05 indicates significant differences between the -Zn and CK treatments. | | | | | | | | | | | | | |

| **TABLE S2** Zn concentrations of shoot and root in the -Zn and CK treatments | | | | | | | |
| --- | --- | --- | --- | --- | --- | --- | --- |
| Inbred  lines | Shoot Zn concentration (μg g^-1^) | | |  | Root Zn concentration (μg g^-1^) | | |
|  | -Zn ^a^ | CK | *P* ^b^ |  | - Zn | CK | *P* |
| Ye478 | 13.1 abcd | 39.1 bc | 0.001 |  | 24.5 efg | 93.8 cd | 0.001 |
| CI7 | 15.3 abcd | 49.5 a | 0.000 |  | 30.0 cdef | 112.2 bc | 0.000 |
| Yu87-1 | 15.7 abcd | 39.2 bc | 0.003 |  | 29.9 cdef | 92.9 cd | 0.001 |
| DE3 | 12.0 bcd | 39.0 bc | 0.003 |  | 17.9 g | 76.6 defg | 0.000 |
| By815 | 9.2 d | 23.5 ef | 0.000 |  | 38.7 bcd | 85.5 def | 0.001 |
| Zheng58 | 10.8 cd | 18.2 f | 0.015 |  | 34.8 bcde | 78.8 defg | 0.000 |
| KUI3 | 15.0 abcd | 27.8 def | 0.001 |  | 25.9 efg | 57.9 g | 0.001 |
| B77 | 12.8 bcd | 36.0 bcd | 0.000 |  | 28.9 cdefg | 85.3 def | 0.006 |
| SC55 | 12.4 bcd | 31.8 cde | 0.000 |  | 38.7 bcd | 125.4 b | 0.000 |
| SK | 19.1 ab | 30.7 cde | 0.003 |  | 35.8 bcde | 56.5 g | 0.007 |
| By804 | 7.9 d | 23.3 ef | 0.002 |  | 22.4 fg | 86.9 def | 0.000 |
| Dan340 | 17.8 abc | 26.0 def | 0.045 |  | 27.1 defg | 76.1 defg | 0.000 |
| Chang7-2 | 14.4 abcd | 45.2 ab | 0.001 |  | 43.7 b | 90.7 cde | 0.000 |
| X178 | 10.1 cd | 28.9 cde | 0.000 |  | 24.6 efg | 86.2 def | 0.000 |
| Mo17 | 14.4 abcd | 32.4 cde | 0.000 |  | 28.9 cdefg | 68.1 efg | 0.000 |
| Zong3 | 11.2 cd | 30.9 cde | 0.000 |  | 17.6 g | 65.1 fg | 0.001 |
| B73 | 10.7 cd | 25.3 def | 0.000 |  | 40.5 bc | 155.0 a | 0.000 |
| HuangC | 15.1 abcd | 39.0 bc | 0.000 |  | 30.1 cdef | 95.8 cd | 0.001 |
| K22 | 20.4 a | 26.6 def | 0.058 |  | 57.5 a | 164.7 a | 0.000 |
| Wu312 | 9.1 d | 27.1 def | 0.005 |  | 29.1 cdefg | 33.8 h | 0.102 |
| a Different letters indicate significant differences among twenty maize inbred lines in the -Zn or CK treatment at *P* ˂ 0.05. | | | | | | | |
| b *P* ˂ 0.05 indicates significant differences between the -Zn and CK treatments. | | | | | | | |

| **TABLE S3** Zn contents of shoot and root in the -Zn and CK treatments | | | | | | | |
| --- | --- | --- | --- | --- | --- | --- | --- |
| Inbred  lines | Shoot Zn content (μg plant^-1^) | | |  | Root Zn content (μg plant^-1^) | | |
|  | -Zn ^a^ | CK | *P* ^b^ |  | - Zn | CK | *P* |
| Ye478 | 49.9 b | 119.7 bc | 0.043 |  | 24.1 b | 63.2 bcde | 0.007 |
| CI7 | 68.4 a | 190.4 a | 0.000 |  | 39.2 a | 109.5 a | 0.001 |
| Yu87-1 | 30.5 c | 70.1 cde | 0.032 |  | 17.3 bcd | 40.6 defghi | 0.010 |
| DE3 | 13.6 defg | 61.4 de | 0.013 |  | 6.3 e | 26.0 ghi | 0.019 |
| By815 | 22.1 cdef | 56.4 de | 0.001 |  | 41.6 a | 66.9 bcde | 0.082 |
| Zheng58 | 26.4 cd | 57.4 de | 0.009 |  | 24.7 b | 61.6 bcdef | 0.013 |
| KUI3 | 22.9 cdef | 45.6 de | 0.027 |  | 11.1 cde | 26.2 ghi | 0.002 |
| B77 | 32.2 c | 112.5 bc | 0.001 |  | 21.2 bc | 68.8 bcd | 0.006 |
| SC55 | 19.9 cdefg | 93.1 bcd | 0.000 |  | 15.2 bcde | 74.6 bcd | 0.000 |
| SK | 10.5 fg | 24.3 e | 0.005 |  | 6.6 e | 11.6 i | 0.001 |
| By804 | 20.8 cdef | 88.1 bcd | 0.000 |  | 20.8 bc | 89.4 ab | 0.002 |
| Dan340 | 21.8 cdef | 59.6 de | 0.007 |  | 12.7 cde | 41.0 defghi | 0.012 |
| Chang7-2 | 17.8 cdefg | 88.3 bcd | 0.001 |  | 11.1 cde | 28.0 fghi | 0.008 |
| X178 | 10.9 efg | 49.7 de | 0.003 |  | 11.9 cde | 46.5 cdefgh | 0.001 |
| Mo17 | 13.8 defg | 52.3 de | 0.006 |  | 8.5 de | 32.5 efghi | 0.021 |
| Zong3 | 25.6 cde | 127.2 b | 0.000 |  | 10.3 de | 69.8 bcd | 0.002 |
| B73 | 15.3 defg | 59.8 de | 0.018 |  | 12.5 cde | 78.2 bc | 0.001 |
| HuangC | 12.1 defg | 81.0 bcd | 0.000 |  | 5.5 e | 53.0 cdefg | 0.008 |
| K22 | 15.0 defg | 48.0 de | 0.000 |  | 15.1 bcde | 76.8 bc | 0.000 |
| Wu312 | 6.2 g | 48.3 de | 0.000 |  | 7.1 de | 16.1 hi | 0.007 |
| a Different letters indicate significant differences among twenty maize inbred lines in the -Zn or CK treatment at *P* ˂ 0.05. | | | | | | | |
| b *P* ˂ 0.05 indicates significant difference between the -Zn and CK treatments. | | | | | | | |

| **TABLE S4** Zn uptake efficiency and Zn relative transport in the -Zn and CK treatments | | | | | | | |
| --- | --- | --- | --- | --- | --- | --- | --- |
| Inbred  lines | Zn uptake (μg g^-1^ root dry weight) | | |  | Zn relative transport (%) | | |
|  | -Zn ^a^ | CK | *P* ^b^ |  | -Zn | CK | *P* |
| Ye478 | 75.2 bcdef | 270.7 bc | 0.009 |  | 67.4 abc | 65.1 bcd | 0.226 |
| CI7 | 82.9 abcde | 309.7 ab | 0.001 |  | 63.5 abc | 63.6 cd | 0.487 |
| Yu87-1 | 82.3 abcde | 249.6 bcde | 0.000 |  | 63.4 abc | 62.9 cd | 0.439 |
| DE3 | 57.0 def | 295.5 b | 0.003 |  | 68.6 ab | 69.8 abc | 0.388 |
| By815 | 59.3 def | 159.7 ef | 0.002 |  | 34.6 h | 46.2 ghi | 0.016 |
| Zheng58 | 66.7 cdef | 152.8 f | 0.002 |  | 51.7 defg | 48.4 ghi | 0.059 |
| KUI3 | 79.6 abcdef | 175.4 def | 0.002 |  | 67.3 abc | 67.0 abc | 0.383 |
| B77 | 72.5 bcdef | 224.7 bcdef | 0.001 |  | 60.1 abcde | 62.1 cd | 0.299 |
| SC55 | 83.0 abcde | 289.9 b | 0.001 |  | 56.7 cdefg | 55.5 defg | 0.328 |
| SK | 98.1 abc | 164.3 ef | 0.003 |  | 61.2 abcd | 67.6 abc | 0.080 |
| By804 | 44.9 f | 173.4 def | 0.000 |  | 49.9 efg | 49.8 fgh | 0.487 |
| Dan340 | 72.4 bcdef | 180.8 cdef | 0.003 |  | 63.9 abc | 59.3 cdef | 0.189 |
| Chang7-2 | 108.0 ab | 379.3 a | 0.006 |  | 58.0 bcdef | 75.8 a | 0.019 |
| X178 | 47.3 ef | 177.9 def | 0.000 |  | 48.1 fg | 51.4 efgh | 0.063 |
| Mo17 | 75.3 bcdef | 181.0 cdef | 0.000 |  | 61.6 abcd | 62.3 cd | 0.415 |
| Zong3 | 56.1 def | 183.7 cdef | 0.001 |  | 69.4 a | 64.5 cd | 0.067 |
| B73 | 83.0 abcde | 272.4 bc | 0.000 |  | 50.9 defg | 43.0 hi | 0.021 |
| HuangC | 87.0 abcd | 261.0 bcd | 0.001 |  | 68.9 ab | 60.6 cde | 0.041 |
| K22 | 114.1 a | 269.3 bc | 0.000 |  | 49.6 efg | 38.7 i | 0.007 |
| Wu312 | 54.3 def | 135.7 f | 0.007 |  | 46.5 g | 75.0 ab | 0.000 |
| a Different letters indicate significant differences among twenty maize inbred lines in the -Zn or CK treatment at *P* ˂ 0.05. | | | | | | | |
| b *P* ˂ 0.05 indicates significant differences between the -Zn and CK treatments. | | | | | | | |
|  | | | | | | | |

| **TABLE S5** Shoot concentrations of Fe, Mn and Cu in the -Zn and CK treatments | | | | | | | | | | | |
| --- | --- | --- | --- | --- | --- | --- | --- | --- | --- | --- | --- |
| Inbred  lines | Shoot Fe concentration (μg g^-1^) | | |  | Shoot Mn concentration (μg g^-1^) | | |  | Shoot Cu concentration (μg g^-1^) | | |
|  | -Zn ^a^ | CK | *P* ^b^ |  | -Zn | CK | *P* |  | -Zn | CK | *P* |
| Ye478 | 102.7 cd | 70.1 ef | 0.009 |  | 246.2 a | 206.3 a | 0.081 |  | 18.2 cd | 11.4 efgh | 0.033 |
| CI7 | 93.7 cd | 67.8 ef | 0.032 |  | 192.3 bc | 214.4 a | 0.249 |  | 15.9 d | 13.4 bcdef | 0.186 |
| Yu87-1 | 129.6 bcd | 87.4 abcde | 0.019 |  | 139.9 cdefg | 111.9 defgh | 0.109 |  | 15.2 d | 9.2 hi | 0.045 |
| DE3 | 114.6 bcd | 76.6 cdef | 0.001 |  | 126.0 defgh | 114.4 cdefgh | 0.041 |  | 19.3 cd | 13.7 bcdef | 0.000 |
| By815 | 108.4 cd | 98.3 abcd | 0.244 |  | 95.0 fghi | 90.4 ghi | 0.124 |  | 31.8 ab | 20.6 a | 0.011 |
| Zheng58 | 126.9 bcd | 68.9 ef | 0.006 |  | 150.4 bcdef | 92.8 fghi | 0.021 |  | 16.8 cd | 9.7 ghi | 0.020 |
| KUI3 | 116.6 bcd | 92.1 abcde | 0.018 |  | 165.6 bcd | 153.0 bc | 0.089 |  | 22.4 cd | 15.8 bc | 0.001 |
| B77 | 100.9 cd | 68.0 ef | 0.061 |  | 148.2 bcdef | 143.7 bcde | 0.357 |  | 22.5 cd | 14.9 bcde | 0.019 |
| SC55 | 111.4 cd | 74.5 cdef | 0.043 |  | 136.8 cdefg | 131.6 bcdef | 0.416 |  | 18.7 cd | 13.2 bcdef | 0.025 |
| SK | 134.7 bc | 110.2 a | 0.015 |  | 199.9 b | 210.0 a | 0.214 |  | 22.8 cd | 16.7 b | 0.014 |
| By804 | 160.8 b | 73.7 cdef | 0.004 |  | 164.9 bcd | 128.0 bcdefg | 0.021 |  | 38.3 a | 22.3 a | 0.015 |
| Dan340 | 119.8 bcd | 106.6 ab | 0.159 |  | 155.9 bcde | 130.7 bcdef | 0.054 |  | 25.3 bc | 15.2 bcd | 0.001 |
| Chang7-2 | 80.7 d | 90.4 abcde | 0.047 |  | 103.6 efghi | 104.5 efghi | 0.471 |  | 14.8 d | 11.9 defgh | 0.008 |
| X178 | 126.6 bcd | 94.5 abcde | 0.014 |  | 125.8 defgh | 121.2 bcdefgh | 0.249 |  | 32.4 ab | 21.3 a | 0.000 |
| Mo17 | 120.5 bcd | 80.9 bcdef | 0.004 |  | 86.6 ghi | 83.1 hi | 0.325 |  | 17.7 cd | 13.6 bcdef | 0.019 |
| Zong3 | 86.4 cd | 59.4 f | 0.015 |  | 64.4 i | 68.6 i | 0.308 |  | 16.0 d | 11.1 fgh | 0.010 |
| B73 | 130.3 bcd | 71.7def | 0.010 |  | 97.4 fghi | 95.4 fghi | 0.372 |  | 20.9 cd | 12.8 cdefg | 0.003 |
| HuangC | 111.8 cd | 93.1 abcde | 0.037 |  | 138.4 cdefg | 157.0 b | 0.004 |  | 19.0 cd | 16.3 bc | 0.008 |
| K22 | 221.7 a | 101.1 abc | 0.001 |  | 196.6 b | 145.8 bcd | 0.007 |  | 33.8 a | 15.6 bc | 0.000 |
| Wu312 | 99.2 cd | 68.9 ef | 0.054 |  | 72.1 hi | 66.3 i | 0.335 |  | 16.5 d | 7.5 i | 0.015 |
| a Different letters indicate significant differences among twenty maize inbred lines in the -Zn or CK treatment at *P* ˂ 0.05. | | | | | | | | | | | |
| b *P* ˂ 0.05 indicates significant differences between the -Zn and CK treatments. | | | | | | | | | | | |

| **TABLE S6** Root concentrations of Mn and Cu in the -Zn and CK treatments | | | | | | | |
| --- | --- | --- | --- | --- | --- | --- | --- |
| Inbred  lines | Root Mn concentration (μg g^-1^) | | |  | Root Cu concentration (μg g^-1^) | | |
|  | -Zn ^a^ | CK | *P* ^b^ |  | -Zn | CK | *P* |
| Ye478 | 807.5 bc | 528.3 fghi | 0.018 |  | 95.4 fg | 44.1 e | 0.005 |
| CI7 | 1092.2 ab | 1626.5 a | 0.037 |  | 168.3 bcd | 147.3 b | 0.138 |
| Yu87-1 | 1038.0 ab | 1191.5 abc | 0.280 |  | 175.5 bcd | 146.5 b | 0.204 |
| DE3 | 804.6 bc | 868.6 cdef | 0.038 |  | 126.7 def | 105.3 c | 0.091 |
| By815 | 282.2 de | 379.3 ghi | 0.072 |  | 114.6 ef | 68.4 de | 0.003 |
| Zheng58 | 311.7 de | 692.8 defgh | 0.005 |  | 170.0 bcd | 122.4 bc | 0.021 |
| KUI3 | 490.2 cde | 601.7 efgh | 0.136 |  | 86.6 fg | 42.3 e | 0.003 |
| B77 | 289.5 de | 306.9 ghi | 0.243 |  | 153.8 cde | 110.0 bc | 0.014 |
| SC55 | 1232.9 a | 1622.4 a | 0.102 |  | 135.4 def | 97.5 cd | 0.012 |
| SK | 566.1 cd | 1056.7 cde | 0.014 |  | 194.2 abc | 111.6 bc | 0.008 |
| By804 | 625.3 cd | 734.4 cdefg | 0.002 |  | 237.2 a | 95.3 cd | 0.000 |
| Dan340 | 443.5 cde | 1122.8 bcd | 0.000 |  | 232.2 a | 192.5 a | 0.014 |
| Chang7-2 | 175.8 e | 368.5 ghi | 0.019 |  | 98.7 fg | 133.0 bc | 0.013 |
| X178 | 468.2 cde | 904.2 cdef | 0.008 |  | 202.4 abc | 131.3 bc | 0.003 |
| Mo17 | 253.9 de | 211.6 hi | 0.083 |  | 214.3 ab | 117.1 bc | 0.000 |
| Zong3 | 264.2 de | 490.1 fghi | 0.030 |  | 101.7 fg | 68.0 de | 0.016 |
| B73 | 1050.4 ab | 1539.9 ab | 0.004 |  | 151.7 cde | 117.0 bc | 0.015 |
| HuangC | 630.0 cd | 580.2 fgh | 0.011 |  | 83.8 fg | 58.6 e | 0.034 |
| K22 | 535.0 cde | 1138.1 bcd | 0.003 |  | 164.2 bcde | 125.4 bc | 0.000 |
| Wu312 | 272.1 de | 87.5 i | 0.006 |  | 60.7 g | 34.5 e | 0.076 |
| a Different letters indicate significant differences among twenty maize inbred lines in the -Zn or CK treatment at *P* ˂ 0.05. | | | | | | | |
| b *P* ˂ 0.05 indicates significant differences between the -Zn and CK treatments. | | | | | | | |
|  | | | | | | | |

| **TABLE S7** Uptake efficiency of Fe, Mn and Cu in the -Zn and CK treatments | | | | | | | | | | | |
| --- | --- | --- | --- | --- | --- | --- | --- | --- | --- | --- | --- |
| Inbred  lines | Fe (μg g^-1^ root dry weight) | | |  | Mn (μg g^-1^ root dry weight) | | |  | Cu (μg g^-1^ root dry weight) | | |
|  | -Zn ^a^ | CK | *P* ^b^ |  | -Zn | CK | *P* |  | -Zn | CK | *P* |
| Ye478 | 2635.4 e | 3652.1 de | 0.093 |  | 1765.1 ab | 1466.8 bcdef | 0.132 |  | 166.6 efg | 95.2 fg | 0.005 |
| CI7 | 5001.0 bcde | 6032.9 ab | 0.001 |  | 1921.4 a | 2481.8 a | 0.085 |  | 221.9 cdef | 200.9 bc | 0.140 |
| Yu87-1 | 4042.4 bcde | 2813.6 ef | 0.030 |  | 1506.6 abc | 1655.0 bcde | 0.347 |  | 214.8 cdef | 183.4 bc | 0.163 |
| DE3 | 4568.6 bcde | 3478.6 de | 0.155 |  | 1132.6 cde | 1383.8 bcdef | 0.050 |  | 190.1 def | 158.1 cde | 0.044 |
| By815 | 3840.3 cde | 2825.3 ef | 0.095 |  | 511.7 f | 664.5 ghi | 0.022 |  | 191.3 def | 133.4 def | 0.013 |
| Zheng58 | 3692.4 cde | 3618.9 de | 0.452 |  | 863.8 ef | 1070.0 efgh | 0.077 |  | 250.4 bcd | 161.7 bcde | 0.031 |
| KUI3 | 4285.5 bcde | 3490.7 de | 0.070 |  | 1083.1 cde | 1316.9 cdef | 0.003 |  | 166.8 efg | 114.9 f | 0.002 |
| B77 | 4372.2 bcde | 2428.6 efg | 0.085 |  | 793.5 ef | 863.4 fghi | 0.178 |  | 230.2 cde | 167.8 bcde | 0.012 |
| SC55 | 4095.8 bcde | 4412.1 cd | 0.235 |  | 1489.5 abc | 2491.5 a | 0.000 |  | 203.7 cdef | 161.2 bcde | 0.016 |
| SK | 6725.6 ab | 6995.5 a | 0.409 |  | 1183.0 cde | 1844.6 bc | 0.027 |  | 266.8 bc | 170.0 bcde | 0.012 |
| By804 | 5182.2 bcde | 2837.6 ef | 0.029 |  | 1093.2 cde | 1210.1 defg | 0.035 |  | 346.1 a | 178.1 bcd | 0.001 |
| Dan340 | 5519.6 bcd | 3426.2 def | 0.044 |  | 919.2 def | 1649.3 bcde | 0.000 |  | 309.4 ab | 253.9 a | 0.003 |
| Chang7-2 | 3411.7 cde | 2449.2 efg | 0.113 |  | 693.9 ef | 923.0 fghi | 0.026 |  | 174.0 efg | 208.9 b | 0.069 |
| X178 | 5753.4 bc | 5113.0 bc | 0.116 |  | 752.3 ef | 1287.9 cdef | 0.006 |  | 249.7 bcd | 198.8 bc | 0.053 |
| Mo17 | 4139.2 bcde | 2552.8 efg | 0.000 |  | 533.4 f | 501.2 hi | 0.239 |  | 271.4 bc | 164.9 bcde | 0.000 |
| Zong3 | 2531.8 e | 1938.0 fg | 0.063 |  | 542.2 f | 851.6 fghi | 0.045 |  | 156.7 fg | 110.7 f | 0.014 |
| B73 | 3903.8 cde | 2889.0 ef | 0.135 |  | 1398.5 bcd | 1981.2 ab | 0.003 |  | 226.5 cdef | 176.4 bcd | 0.000 |
| HuangC | 3717.7 cde | 3400.2 def | 0.247 |  | 1116.6 cde | 1134.1 defg | 0.459 |  | 164.2 efg | 123.9 ef | 0.003 |
| K22 | 8417.9 a | 5418.3 bc | 0.001 |  | 1081.2 cde | 1711.9 bcd | 0.003 |  | 258.2 bcd | 187.0 bc | 0.000 |
| Wu312 | 2851.3 de | 1324.7 g | 0.001 |  | 473.2 f | 342.2 i | 0.101 |  | 106.9 g | 63.4 g | 0.059 |
| a Different letters indicate significant differences among twenty maize inbred lines in the -Zn or CK treatment at *P* ˂0.05. | | | | | | | | | | | |
| b *P* ˂ 0.05 indicates significant differences between the -Zn and CK treatments. | | | | | | | | | | | |

| **TABLE S8** Relative transport (%) of Fe, Mn and Cu in the -Zn and CK treatments | | | | | | | | |
| --- | --- | --- | --- | --- | --- | --- | --- | --- |
| Inbred  lines |  | Mn transport (%) | | |  | Cu transport (%) | | |
|  |  | -Zn ^a^ | CK | *P* ^b^ |  | -Zn | CK | *P* |
| Ye478 |  | 54.3 bc | 63.6 ab | 0.057 |  | 42.6 ab | 53.7 ab | 0.063 |
| CI7 |  | 34.2 efg | 34.4 efg | 0.452 |  | 24.5 def | 26.5 fgh | 0.294 |
| Yu87-1 |  | 31.1 efg | 27.7 fg | 0.167 |  | 18.5 f | 20.7 h | 0.379 |
| DE3 |  | 36.8 defg | 32.0 efg | 0.104 |  | 33.6 bcde | 33.4 defgh | 0.471 |
| By815 |  | 45.0 cde | 43.2 cde | 0.369 |  | 40.0 abc | 48.4 bc | 0.043 |
| Zheng58 |  | 55.0 bc | 35.3 efg | 0.011 |  | 21.2 ef | 24.4 gh | 0.167 |
| KUI3 |  | 54.8 bc | 54.3 bcd | 0.468 |  | 48.1 a | 63.1 a | 0.027 |
| B77 |  | 63.5 ab | 64.4 ab | 0.246 |  | 33.2 bcde | 34.5 defg | 0.058 |
| SC55 |  | 28.6 fg | 28.5 efg | 0.478 |  | 33.4 bcde | 39.6 cdef | 0.093 |
| SK |  | 52.2 bcd | 41.4 def | 0.051 |  | 27.5 cdef | 35.7 cdefg | 0.144 |
| By804 |  | 42.8 cdef | 39.2 ef | 0.099 |  | 31.3 bcdef | 46.5 bcd | 0.006 |
| Dan340 |  | 52.0 bcd | 31.8 efg | 0.001 |  | 25.0 def | 24.2 gh | 0.315 |
| Chang7-2 |  | 76.0 a | 64.2 ab | 0.112 |  | 43.9 ab | 36.2 cdefg | 0.085 |
| X178 |  | 38.5 cdefg | 29.9 efg | 0.062 |  | 30.1 bcdef | 34.0 defg | 0.219 |
| Mo17 |  | 52.5 bcd | 57.8 b | 0.047 |  | 21.0 ef | 29.0 fgh | 0.028 |
| Zong3 |  | 41.6 cdef | 31.6 efg | 0.032 |  | 35.2 abcde | 38.9 cdef | 0.061 |
| B73 |  | 25.0 g | 22.3 g | 0.156 |  | 33.1 bcde | 33.7 defgh | 0.452 |
| HuangC |  | 53.6 bc | 55.9 bc | 0.371 |  | 49.1 abcd | 52.8 ab | 0.237 |
| K22 |  | 51.6 bcd | 33.5 efg | 0.015 |  | 36.4 a | 32.9 efgh | 0.016 |
| Wu312 |  | 42.6 cdef | 73.9 a | 0.006 |  | 43.7 ab | 44.6 bcde | 0.466 |
| a Different letters indicate significant differences among twenty maize inbred lines in the -Zn or CK treatment at *P* ˂ 0.05. | | | | | | | | |
| b *P* ˂ 0.05 indicates significant differences between the -Zn and CK treatments. | | | | | | | | |

| **TABLE** **S9** Spearman correlation coefficients (*r*) between Zn score and SDW, RDW, R/S in the -Zn and -Zn/CK treatments | |
| --- | --- |
| Traits | Zn score |
| SDW (-Zn) | 0.657** |
| RDW (-Zn) | 0.332** |
| R/S (-Zn) | -0.652** |
| SDW (-Zn/CK) | 0.559** |
| RDW (-Zn/CK) | 0.289** |
| R/S (-Zn/CK) | -0.512** |
| ** indicates significant differences between the -Zn and CK treatments at *P* ˂ 0.01. | |

| **TABLE S10** Pearson correlation coefficients between the biomass traits under different treatments | | | |
| --- | --- | --- | --- |
|  | SDW (-Zn) | SDW (CK) | RDW (-Zn/CK) |
| RDW (-Zn) | 0.811** | NS | 0.638** |
| RDW (CK) | NS | 0.772** | -0.302** |
| SDW (-Zn/CK) | 0.766** | -0.170** | 0.674** |
| NS represents no significance. ** indicates significant difference at *P <* 0.05. | | | |

| **TABLE S11** The primers used for Real-time PCR | | | |
| --- | --- | --- | --- |
| Gene name | NCBI sequence | Primer | Sequence 5' to 3' |
| *ZmZIP1* | NM_001137726 | ZmZIP1F | CCTCTCTGCGTTGGTTGCTCT |
|  |  | ZmZIP1R | TTGATGGTTGTTTTCTGGTCGT |
| *ZmZIP2* | NM_001159169 | ZmZIP2F | CCACAAATGGCACGAGGTCT |
|  |  | ZmZIP2R | CGAAGACGGAGTGGAAGCAAA |
| *ZmZIP3* | NM_001155536 | ZmZIP3F | GCCTCTTGTTGGTGCCCTTA |
|  |  | ZmZIP3R | TCAACAATGAACGCTGTAGTGCT |
| *ZmZIP4* | HM048832 | ZmZIP4F | CCTTCTTCTCGCTCACCGCT |
|  |  | ZmZIP4R | AGCCTCGGGTTGCTGAAGT |
| *ZmZIP5* | NM_001154257 | ZmZIP5F | GCACATAGGCATAGCCACGC |
|  |  | ZmZIP5R | ACGCCCAAAGATAGCCCGAT |
| *ZmZIP6* | NM_001156151 | ZmZIP6F | ATCAGGTGTTCGAGGGGATG |
|  |  | ZIP6R | TGCTATCGTCGTAGCCAGTC |
| *ZmZIP7* | NM_001157018 | ZmZIP7F | ACTAGGTGGGTGCATTGCTCAG |
|  |  | ZmZIP7R | TGCCAGCAGATACCGAGTCAA |
| *ZmZIP8* | NM_001154769 | ZmZIP8F | CGTGTCATCGCTCAGGTTCTTG |
|  |  | ZmZIP8R | CCCTCGAACATTTGGTGGAAG |
| (Li et al., 2013) | | | |
